# The class VIII myosin ATM1 is required for root apical meristem function

**DOI:** 10.1242/dev.201762

**Published:** 2023-07-04

**Authors:** Damilola Olatunji, Natalie M. Clark, Dior R. Kelley

**Affiliations:** ^1^ Department of Genetics, Development and Cell Biology, Iowa State University, Ames, IA 50011, USA; ^2^ Broad Institute, Cambridge, MA 04212, USA

**Keywords:** Auxin, Cell proliferation, Myosin, Root apical meristem, Sugar, *Arabidopsis thaliana*

## Abstract

Myosins are evolutionarily conserved motor proteins that interact with actin filaments to regulate organelle transport, cytoplasmic streaming and cell growth. Plant-specific class XI myosin proteins direct cell division and root organogenesis. However, the roles of plant-specific class VIII myosin proteins in plant growth and development are less understood. Here, we investigated the function of an auxin-regulated class VIII myosin, *Arabidopsis thaliana* MYOSIN 1 (ATM1), using genetics, transcriptomics and live cell microscopy. ATM1 is associated with the plasma membrane and plasmodesmata within the root apical meristem (RAM). Loss of ATM1 function results in decreased RAM size and reduced cell proliferation in a sugar-dependent manner. Auxin signaling and transcriptional responses were dampened in *atm1-1* roots. Complementation of *atm1-1* with a tagged ATM1 driven under the native *ATM1* promoter restored root growth and cell cycle progression. Genetic analyses of *atm1-1* seedlings with HEXOKINASE 1 (HXK1) and TARGET OF RAPAMYCIN COMPLEX 1 (TORC1) overexpression lines indicate that ATM1 is downstream of TOR. Collectively, these results provide previously unreported evidence that ATM1 functions to influence cell proliferation in primary roots in response to auxin and sugar cues.

## Teaser

**Summary:** An auxin-regulated class VIII myosin, ATM1, is required for root cell proliferation in response to sugar.

## INTRODUCTION

In multicellular organisms, organogenesis relies on the coordination of three interwoven processes: cell proliferation, elongation and differentiation (De Smet and Beeckman, 2011; Harashima and Schnittger, 2010; Stals and Inzé, 2001; Zluhan-Martínez et al., 2021). Cell proliferation events within a developing organ drive growth via an increase in cell number that is triggered and sustained by growth cues, which is a tightly regulated process (Polymenis and Aramayo, 2015). In contrast, cell elongation contributes to cell size regulation, and as a consequence can fine tune organismal body plan (De Cnodder et al., 2006; Marshall et al., 2012; Szövényi et al., 2019). During cell differentiation, a variety of cell types constituting a tissue and/or organ become specialized, with distinct patterns and forms (Hulskamp et al., 1998). Organogenesis in plants is predominantly post-embryonic and relies on stem cell niches in the shoot and root apical meristems to continuously produce new cells that assume specific differentiation patterns (Burian et al., 2016; Shishkova et al., 2008). Roots are an ideal model to study the coordination of these processes because a growing primary root contains: (1) a meristematic zone, harboring active dividing cells; (2) a transition zone (TZ), found between the basal meristem and the meristematic zone, containing cells that still have the competence to divide but at relatively slow rate; (3) an elongation zone (EZ), which has cells with an accelerated expansion rate, mainly in length but not in width; and (4) a differentiation zone (DZ), which has cells that have stopped expansion, but begin to differentiate into specialized tissues. Phytohormones are the major regulator of plant growth responses and a great amount of information has been generated on this (Lv et al., 2019; Santner et al., 2009; Shi and Vernoux, 2022); however, the role of sugars as growth cues is now receiving a great deal of attention. Therefore, understanding the mechanisms of how root organogenesis is regulated in response to both hormonal and metabolic signals is important for furthering our understanding of plant growth and development.

As the by-product of photosynthesis, sugars are translocated to sink organs including roots to orchestrate growth and branching programs, mostly in the form of sucrose, which is subsequently converted to glucose and fructose for the sustenance of energy metabolism (Kaur et al., 2021; Li and Sheen, 2016; Rolland et al., 2006). Genetic and biochemical studies have revealed the link between plant TARGET OF RAPAMYCIN (TOR) and nutrient signaling (Dong et al., 2015; Xiong et al., 2013). TOR is recognized as TOR COMPLEX 1 (TORC1), sharing related structural organization with budding yeast and animals. Typically, this complex consists of TOR and its interacting partners: REGULATORY-ASSOCIATED PROTEIN OF TOR (RAPTOR) and LETHAL WITH SEC THIRTEEN 8 (LST8) (Burkart and Brandizzi, 2020; Dobrenel et al., 2016; Moreau et al., 2012). Sugar availability is the main activator of TOR kinase in plants resulting in the control of cell proliferation and reprogramming of the transcriptome of the meristems (Xiong et al., 2013). In the presence of glucose, TOR is activated and directly phosphorylates E2FA/B transcription factors to maintain the shoot and root meristematic activities (Li et al., 2017). Many of the downstream players in this process are not well understood and it is unknown how stem cell properties are modulated in response to sugars.

Recent studies have revealed the contributions of plant myosins in the regulation of plant growth and developmental programs in response to carbon and hormonal cues (Abu-Abied et al., 2018; Han et al., 2021 preprint; Holweg, 2007; Holweg et al., 2003; Ojangu et al., 2018; Olatunji and Kelley, 2020). In plants, myosins are actin-based motor proteins belonging to myosin VIII and XI families. The *Arabidopsis* genome encodes 13 members of class XI myosins and four members of class VIII motor proteins (Haraguchi et al., 2019; Reddy and Day, 2001; Ryan and Nebenführ, 2018). Myosins of the class XI clade are the most well-studied plant-specific myosins to date and their roles have been implicated in rapid cell growth and expansion (Kurth et al., 2017; Peremyslov et al., 2008, 2010). In contrast, information on the roles of class VIII myosins, including ATM1, in plant development still remains limited. Among plant-specific myosins, ATM1 was the first member to be identified and sequenced (Knight and Kendrick-Jones, 1993). The ortholog of ATM1 in *Physcomitrium patens*, Myosin VIII, is known to play a key role in patterning and cell division in moss (Wu and Benzanilla, 2014; Wu et al., 2011). Immunolocalization studies have shown that ATM1 is localized to the plasmodesmata (PD) and new cell plates in *Arabidopsis* roots (Reichelt et al., 1999). Studies on the full-length or tail domain region of ATM1 fused to green fluorescent protein (GFP) expressed under its native promoter indicated that ATM1 is endogenously localized to the PD, endoplasmic reticulum, plasma membrane, plastids and newly formed cell walls (Golomb et al., 2008; Haraguchi et al., 2014). *ATM1* accumulation was also observed in root and shoot apices using a β-glucuronidase (GUS) reporter transgene (Haraguchi et al., 2014). ATM1 protein levels are increased in response to exogenous indole-3-acetic acid (IAA) treatment (Kelley et al., 2017 preprint). ATM1 plays a key role in sugar-dependent hypocotyl growth, which is driven solely by cell elongation (Olatunji and Kelley, 2020). However, the roles of ATM1 in primary root development are unknown.

Here, we investigated the role of ATM1 in the regulation of root stem cell properties, including proliferation and differentiation. We report that the *atm1-1* mutant exhibits a sugar-dependent short root phenotype due to impaired cell cycle activity. *In situ* DNA labeling and live imaging revealed that ATM1 is required for normal cell proliferation in response to sucrose. Gene expression and auxin response analysis indicate that *atm1-1* root apical meristems exhibit dampened auxin responses in the quiescent center and columella. Transgenic complementation of *atm1-1* mutant with *ATM1pro::GFP-ATM1* or *ATM1pro::GUS-ATM1* cassette can restore root growth and root apical meristem (RAM) activity. In addition, GFP translational reporters for ATM1 expression *in vivo* indicate that this plasma membrane-localized protein is enriched in root stem cell populations. Together these data suggest that ATM1 is required for cell proliferation and differentiation in *Arabidopsis* roots.

## RESULTS

### *ATM1* promoter activity is high in the root apical meristem

**Fig. 1.**
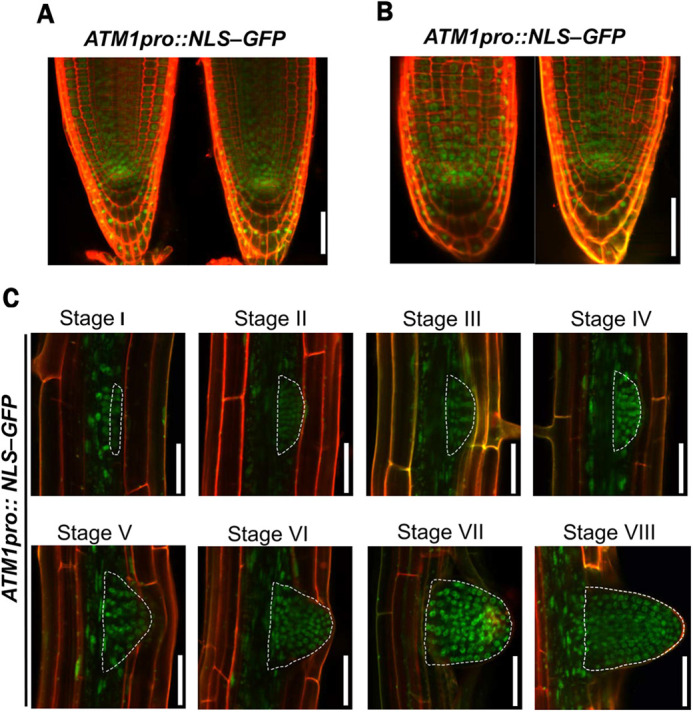
**ATM is expressed during post-embryonic root organogenesis in *Arabidopsis thaliana*.** (A,B) Transcriptional expression pattern of *ATM1* in *Arabidopsis* roots. *ATM1pro::NLS-GFP* construct expression in the stem cell initials of 5-day-old primary roots (A) and in adventitious roots (B). Red, propidium iodide; green, GFP expression. (C) *ATM1* is expressed in all stages of lateral root development, from initiation (stage 1) to emergence (stage VIII). The dashed white lines outline the developing lateral root primordium. Scale bars: 50 µm

To determine ATM1 expression patterns during root development, stable transgenic lines were generated with *ATM1pro::NLS-GFP-GUS* construct consisting of a 4.5 kb *ATM1* promoter sequence. Confocal microcopy of 5-day-old *Arabidopsis* primary roots (Fig. 1A) and adventitious roots (Fig. 1B) revealed that ATM1 is strongly expressed in apical root cells. Because myosin XI has been implicated in post-embryogenic root formation (Abu-Abied et al., 2018), we then monitored ATM1 expression across the eight stages (Stage I-VIII) of lateral roots (LR) formation (Péret et al., 2009). ATM1 expression was uniformly observed in LR primordia at all stages of LR branching programs (Fig. 1C). In addition, histological analysis of GUS activity in *ATM1pro::GUS-ATM1* plants suggests that ATM1 is expressed in regions of active cell division such as the shoot and root apices (Fig. S1A-D). Collectively these expression data demonstrate that ATM1 is expressed in developing roots.

### Loss of *ATM1* results in reduced root growth

**Fig. 2.**
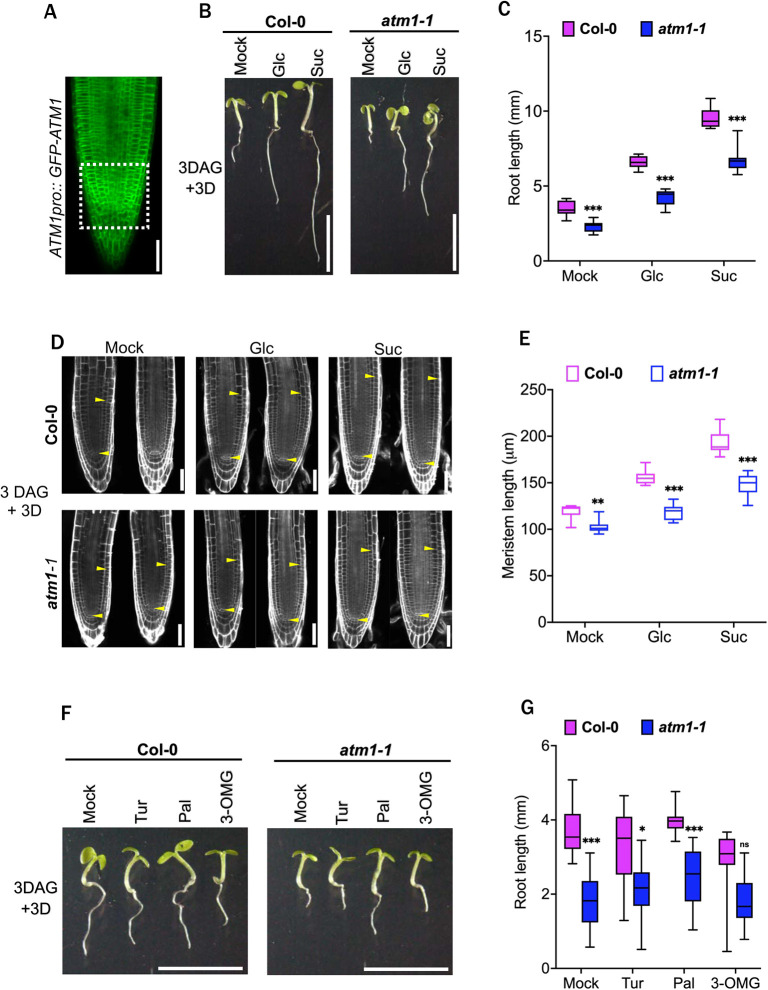
**ATM1 is a plasma membrane-localized protein required for sugar-activated root growth.** (A) GFP-ATM1 protein accumulates at the plasma membrane of root apical meristem cells in 5-day-old plants. The boxed area represents the stem cell niche with elevated GFP-ATM1 accumulation. (B) Re-activation of root growth in 6-day-old wild-type (Col-0) and *atm1-1* seedlings in response to 0.5× MS medium (mock) or supplemented with 15 mM glucose (Glc) or sucrose (Suc) under 15 µmol m^−1^ s^−1^ 12 h light/12 h dark conditions. (C) Quantification of root growth across 3 days following Glc or Suc treatment in Col-0 and *atm1-1* seedlings. *N*=10; ***P<0.0001, two-way ANOVA and Tukey's multiple comparison test. (D) Confocal images of Col-0 and *atm1-1* roots grown without exogenously applied sugar (mock) or with sugars (Glc or Suc) for 3 days. Roots were stained with propidium iodide (PI). Yellow arrowheads delineate the root apical meristem. (E) Root meristem length of 6-day-old Col-0 and *atm1-1* plants. *N*=10; **P<0.01, ***P<0.001, two-way ANOVA and Tukey's multiple comparison test. (F) Phenotypes of 6-day-old Col-0 and *atm1-1* plants grown on 0.5× MS medium with or without 15 mM non-metabolizable sugars (3-OMG, 3-O-methyl-D-glucose; Pal, palatinose; Tur, turanose). (G) Root length of Col-0 and *atm1-1* in response to Tur, Pal and 3-OMG. *N*=10; ns, not significant; *P<0.05, ***P<0.001, two-way ANOVA and Tukey's multiple comparison test. Box plots extend from 25th to 75th percentile; horizontal lines represent median; whiskers represent minimum to maximum values. Scale bars: 50 µm (A,D); 5 mm (B,F).

To examine ATM1 protein expression patterns *in vivo*, stable transgenic lines were generated with *ATM1pro::GFP-ATM1* construct. Confocal microscopy of GFP-ATM1 in *Arabidopsis* roots revealed protein accumulation at the plasma membrane. In addition, this line showed strong GFP-ATM1 accumulation in the meristematic zone of the RAM and stele initials (Fig. 2A). Collectively, these expression data demonstrate that ATM1 is a plasma membrane-localized protein that is present in the primary root.

To genetically investigate the role of ATM1 in plant root development, we used a previously characterized loss-of-function *ATM1* allele (Olatunji and Kelley, 2020), *atm1-1* (SAIL_405_B08). Loss of *ATM1* leads to reduced organ growth in light grown seedlings in the absence of exogenous sugar (Olatunji and Kelley, 2020). Compared with wild-type (WT) Col-0 plants, *atm1-1* root growth was significantly decreased in the absence of exogenous sugar (Fig. 2B,C). Given that ATM1 expression is more pronounced in the region of active cell division in the RAM, we used a previously described root growth re-activation assay (Li et al., 2019; Xiong et al., 2013) to examine how RAM cells respond to sugar signals in *atm1-1*. For this assay, WT and *atm1-1* seedlings were grown without sugar under photosynthesis-constrained low light conditions for 3 days to induce mitotic quiescence. Then, these seedlings were moved to 0.5× Murashige & Skoog (MS) medium (mock) or supplemented with glucose or sucrose to re-activate the arrested root meristems. Compared with WT Col-0, the *atm1-1* roots have reduced sugar-induced growth (Fig. 2B,C).

The sugar-dependent impairment observed in *atm1-1* roots may be due to altered RAM size. To test this idea, we examined the root meristems of 6-day-old plants grown on control liquid 0.5× MS medium (mock) or 0.5× MS supplemented with sugars. The *atm1-1* root meristem length was significantly different from that of WT with or without sugar treatment (Fig. 2D,E). Next, we asked whether the reduced-sugar activated root growth in *atm1-1* is attributed to the function of sugar as energy source or signaling molecules. We analyzed WT and *atm1-1* response to several non-metabolizable sugars: a glucose analog (3-O-methyl-D-glucose; 3-OMG) and two sucrose analogs [palatinose (Pal) and turanose (Tur)]. Three-day-old quiescent seedlings grown on 0.5× MS medium were transferred to growth medium supplemented with 15 mM non-metabolizable sugar and scored for activated root growth after 3 days. When compared with WT, none of the glucose or sucrose analog(s) significantly activates the reduced root growth in *atm1-1* (Fig. 2F,G). Taken together, these results suggest that ATM1 is essential for sugar-dependent root growth and stem cell activity.

### Complementation of *atm1-1* restored root growth to normal

**Fig. 3.**
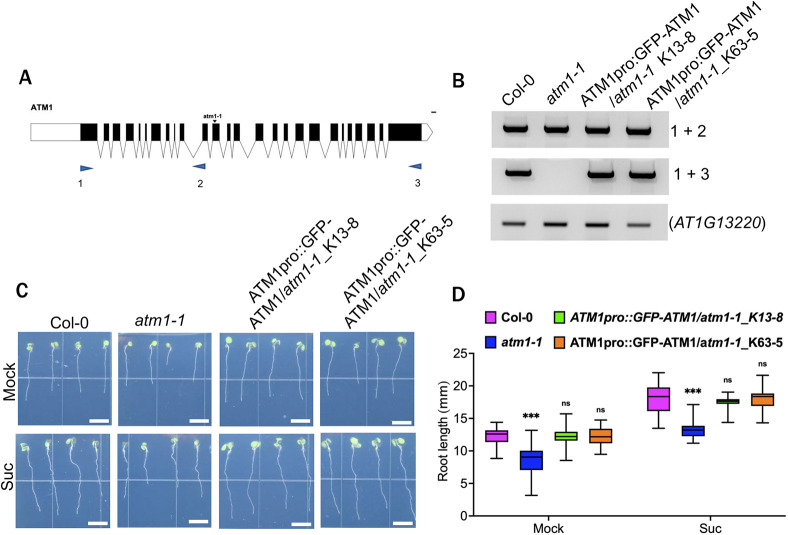
**Complementation of *atm1-1*.** (A) *ATM1* (*AT3G19960.2*) gene model indicating the position of SAIL_405_B08 (*atm1-1*) and primers used for PCR amplification (blue arrows 1-3). (B) RT-PCR of *ATM1* transcript in Col-0, *atm1-1* and complemented lines (*ATM1pro::GFP-ATM1/atm1-1_K13-8* or *ATM1pro::GUS-ATM1/atm1-1_K63-5*). The top panel shows *ATM1* mRNA amplified upstream of the T-DNA insertion site amplified with primers 1+2. The middle panel depicts full-length *ATM1* transcript using primers 1+3. The bottom panel is amplification of a control reference gene *AT1G13220*. (C) Phenotypes of 5-day-old Col-0, *atm1-1* and complimented lines grown on 0.5× MS medium (mock) or supplemented with 15 mM sucrose (Suc) at 45 µmol m^−1^ s^−1^, 12 h light/12 h dark conditions. (D) Root growth in Col-0, *atm1-1* and the two complemented lines. *N*=20; ns, not significant; ***P<0.001, two-way ANOVA and Tukey's multiple comparison test. Box plots extend from 25th to 75th percentile; horizontal lines represent median; whiskers represent minimum to maximum values. Scale bars: 5 mm.

We then asked whether the impaired root growth in the *atm1-1* mutant could be restored. To address this, we cloned the ATM1 full-length genomic sequence, tagged with a GFP or GUS reporter protein at the N-terminal driven by *ATM1* native promoter (a 4.5 kb fragment upstream of the first annotated ATG in the ATM1 coding sequence), and the generated constructs *ATM1pro::GFP-ATM1* was used for complementation. From the T3 transgenic lines, we performed reverse transcriptase-PCR (RT-PCR) with primers designed to amplify *ATM1* transcripts upstream of the site of insertion in *atm1-1* and full-length *ATM1* (Fig. 3A). RT-PCR results showed the expected *ATM1* transcripts in WT, *atm1-1* and *ATM1pro::GFP-ATM1*/*atm1-1* plants upstream of the annotated T-DNA insertion site of *atm1-1* (Fig. 3B). Full-length *ATM1* transcript was not detected in *atm1-1* mutant but was restored to WT levels in the transgene-containing lines (Fig. 3B). Next, we screened the complemented lines for root growth using 5-day-old seedlings grown under our growth conditions. On control plates, the root length of the two complemented lines was not significantly different from that of the WT plants (Fig. 3C,D). Notably, sucrose-induced root growth was restored to WT levels in *ATM1pro::GFP-ATM1*/*atm1-1* lines (Fig. 3C,D). Collectively, these results indicate that a functional ATM1 activity is required for root growth.

### Columella cell differentiation is abnormal in *atm1-1* roots

**Fig. 4.**
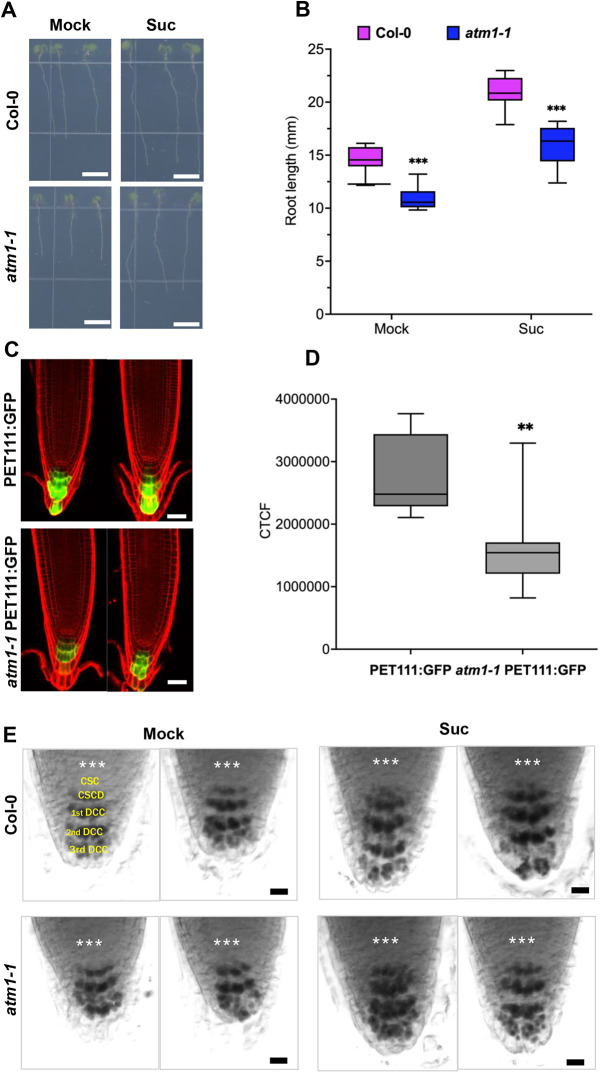
**Loss of ATM1 impacts columella differentiation.** (A) Phenotypes of 5-day-old *atm1-1* plants with reduced root length compared with wild type on 0.5× MS medium without or with 15 mM sucrose (Suc) at 45 µmol m^−1^ s^−1^ 12 h light/12 h long-day conditions. (B) Quantitative analysis of root growth in Col-0 and *atm1-1* seedlings. *N*=10; ***P<0.001, two-way ANOVA and Tukey's multiple correction test. (C) Examination of columella cell marker line *PET111:GFP* accumulation in wild-type and *atm1-1* roots grown on 0.5× MS with 15 mM Suc. Red, propidium iodide; green, GFP expression. (D) Computed average GFP fluorescence intensity in *PET111:GFP* and *atm1-1 PET111:GFP. N*=7-8; **P<0.01, two-way ANOVA and Tukey's multiple comparison test. (E) Starch granule accumulation in Col-0 and *atm1-1* columella cells. Roots of 5-day-old wild type and *atm1-1* plants were stained with Lugol solution before imaging. Asterisks indicate quiescent center. CSC, columella stem cells; CSCD, columella stem cell daughters; 1st DCC, first layer of differentiated columella root cap cells; 2nd DCC, second layer of differentiated columella root cap cells; 3rd DCC, third layer of fully differentiated columella root cap cells. Box plots extend from 25th to 75th percentile; horizontal lines represent median; whiskers represent minimum to maximum values. Scale bars: 5 mm (A); 20 µm (C); 50 µm (E).

To further explore the role of ATM1 in root organogenesis, we examined *atm1-1* roots under normal photosynthetic conditions as previously described (Li et al., 2019). Compared with WT plants, *atm1-1* displayed significantly reduced root growth on sugar-free MS plates (Fig. 4A,B). Moreover, the root growth of *atm1-1* seedlings grown on MS medium supplemented with sucrose was significantly decreased compared with WT plants (Fig. 4A,B). Thus, these data indicate that *ATM1* is required for the regulation of RAM activity. Next, we asked whether the mutation in *ATM1* affects patterning of the root meristem specialized cell types. In *Arabidopsis*, the root cap comprises two distinct cell types: the lateral root cap (LRC) and columella. Distal to the quiescent center (QC) are the LRC and columella stem cells (CSCs). The CSCs give rise to differentiated columella cells (CCs) containing starch granules required for graviperception (Hong et al., 2015; Su et al., 2017). To investigate whether loss of *ATM1* altered the CSC identity, we crossed the previously characterized *PET111:GFP* enhancer trap line (Clark et al., 2019; Nawy et al., 2005) into *atm1-1*. Typically, *PET111:GFP* marks only the differentiated columella cells (Clark et al., 2019; Nawy et al., 2005). Examination of 5-day-old *atm1-1* roots harboring the *PET111:GFP* transgene revealed significantly diminished expression of this columella marker compared with WT in the presence of sucrose (Fig. 4C,D). Next, we asked whether columella stem cell differentiation in *atm1-1* was impaired by proxy of starch granule presence. To address this question, we stained the roots of 5-day-old WT and *atm1-1* seedlings with Lugol solution. Compared with WT, under sugar-free conditions, the layer of columella stem cell daughter cells (CSCDs) was absent in *atm1-1* (Fig. 4E), suggesting that the competence of these cells to properly differentiate is altered. Under sugar supplementation conditions, the size of the CSCDs and the fully differentiated columella cells (DCCs) was reduced in *atm1-1* compared with WT plants, but not the number of cells (Fig. 4E). Despite the mis-expression of the root cap markers in *atm1-1*, GFP expression of the QC marker *WOX5:GFP* is intact in the mutant (Fig. S3). These results are consistent with ATM1 activity being required for normal cell division in the RAM.

### Transcriptomic analysis of *atm1-1* seedlings in response to sugars

**Fig. 5.**
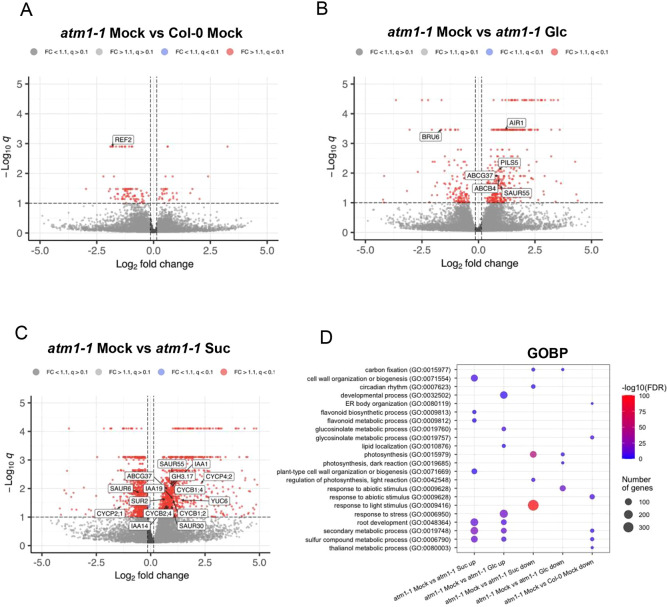
**Transcriptomic analysis of *atm1-1* revealed key biological processes.** (A-C) Volcano plots showing candidate differentially expressed genes (DEGs). (A) DEGs in *atm1-1* compared with Col-0 under mock treatment. (B) DEGs in *atm1-1* in response to glucose treatment. (C) DEGs in *atm1-1* in response to sucrose treatment. (D) Enriched Gene Ontology (GO) Biological Process (GOBP) terms among DEGs in *atm1-1* under different treatment conditions. The color depicts the significance of the enrichment [-log10(FDR)]. The circle size represents the number of genes linked to a specific GO term.

The observed root growth defects in response to both glucose and sucrose in *atm1-1* (Fig. 2), suggested that both glucose and/or sucrose responses may be altered in the mutant. Both sucrose and glucose are well known to regulate gene expression via the distinct and overlapping pathways involving TORC, HEXOKINASE 1 (HXK1; also known as GIN2) and SnRK. In order to determine the gene expression responses to glucose and sucrose in *atm1-1*, we performed bulk RNA-seq analysis using whole seedlings grown with and without exogenously applied sugars (glucose and sucrose) (Table S1). From the transcriptome data, we identified differentially expressed genes (DEGs) that were specifically modulated by sugar molecules in both Col-0 and *atm1-1* mutant compared with mock treatment with significance of *q*-value ≤0.1. Previous studies have examined the effects of sucrose and glucose on transcription in *Arabidopsis* (Mishra et al., 2009; Shulse et al., 2019). In order to uncover sugar-dependent gene regulation that was specifically altered in *atm1-1*, we examined these DEGs in more detail. In total, 32 upregulated DEGs were observed in *atm1-1* compared with WT under mock conditions, whereas *atm1-1* seedlings treated with glucose and sucrose relative to mock samples had 406 and 1488 DEGs, respectively (Fig. 5A-C, right; Table S1). Among these samples, 1154 downregulated DEGs were obtained in *atm1-1* sucrose versus mock samples, followed by glucose-treated *atm1-1* seedlings compared with mock samples (134), and control medium-treated *atm1-1*/WT plants had the lowest number (87) of repressed DEGs (Fig. 5A-C, left; Table S1).

Sucrose-regulated auxin pathway genes in both WT Col-0 and *atm1-1* include numerous SMALL AUXIN UPREGULATED (SAUR) genes, *AUXIN-INDUCED IN ROOT CULTURES 1* (*AIR1*), *AIR3*, *PLEIOTROPIC DRG RESISTANCE 9* (*ABCG37*), and *YADOKARI* (*YDK1*) (Table S1). Notably, in *atm1-1* several classical auxin marker genes are upregulated including *YUCCA 3*, *YUC6*, *YUC8*, *GRETCHEN HAGEN 3.17* (*GH3.17*), *IAA19* and *IAA29* (Fig. 5C; Table S1). These genes are not auxin-upregulated in the WT, suggesting that these gene expression events are due to specific sucrose-dependent cues in the absence of *ATM1*. In addition, these data also suggest that sucrose can both induce and repress many key auxin pathway genes, which is consistent with a previous study (Mishra et al., 2009).

Next, to understand the major molecular processes that are significantly enriched in *atm1-1* upon sugar treatment, we performed gene ontology (GO) analysis. No significantly enriched GO terms were identified for control medium-treated *atm1-1*/WT plant DEGs, but GO terms such as root development, secondary metabolic process and sulfur compound metabolic process were enriched in sucrose- and glucose-treated *atm1-1* samples compared with mock-treated *atm1-1* seedlings, suggesting the essential role of sugar signaling in ATM1-mediated developmental processes (Fig. 5D). Among the enriched GO terms in the downregulated DEGs for *atm1-1* plants fed with sugar molecules are carbon fixation, photosynthesis and response to light stimulus. These results suggest that besides exogenously applied sugars, light-mediated photosynthetic processes are essential for hormones biosynthesis and as carbon sources during ATM1-controlled cell proliferation.

### ATM1 activity is dependent on auxin-sugar signaling

**Fig. 6.**
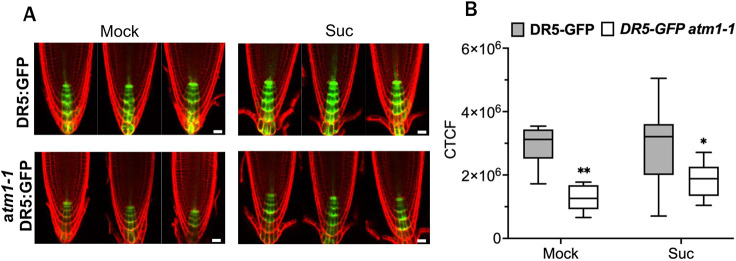
**Loss of *ATM1* alters auxin responses *in planta*.** (A) Expression of an auxin response marker line *DR5:GFP* in Col-0 and *atm1-1*. Roots of 5-day-old seedlings grown on 0.5× MS without or with 15 mM sucrose (Suc) were counterstained with propidium iodide. Red, propidium iodide; green, GFP expression. (B) Calculated corrected total cell fluorescence from *DR5:GFP* and *atm1-1 DR5:GFP* plants. *N*=8-10; *P<0.05, **P<0.01, two-way ANOVA and Tukey's multiple correction test. Box plots extend from 25th to 75th percentile; horizontal lines represent median; whiskers represent minimum to maximum values. Scale bars: 20 µm.

Auxin is a central regulator of root growth, and the roles of multiple auxin biosynthesis, transport and signaling pathways have been implicated in *de novo* root organogenesis in plants (Casimiro et al., 2001; Lavenus et al., 2013; Mishra et al., 2022; Olatunji et al., 2017; Roychoudhry and Kepinski, 2022; Singh et al., 2020). Within the primary root, auxin levels in the QC and columella are associated with columella differentiation (Brumos et al., 2018; Ding and Friml, 2010). Because the transcriptomic analysis of *atm1-1* indicated sucrose-activated gene expression of auxin biosynthesis, metabolism and signaling genes (Fig. 5C; Table S1), and columella marker expression and divisions are altered in *atm1-1* (Fig. 4C,D), we asked whether loss of *ATM1* can impact auxin signaling in the RAM. To examine this *in vivo*, we crossed the previously described auxin response marker *DR5:GFP* (Friml et al., 2003) into *atm1-1*. Confocal imaging of *DR5:GFP* in 5-day-old *atm1-1* roots revealed a significant reduction in auxin response with and without exogenous sugar application compared with WT (Fig. 6A,B), suggesting a downregulation of auxin signaling in the mutant under the experimental conditions tested. Collectively, these data indicate that the spatiotemporal regulation of auxin pathways is impaired in the absence of *ATM1* activity and that this response is linked to sugar during root development.

### Cell cycle activation is impaired in *atm1* roots

**Fig. 7.**
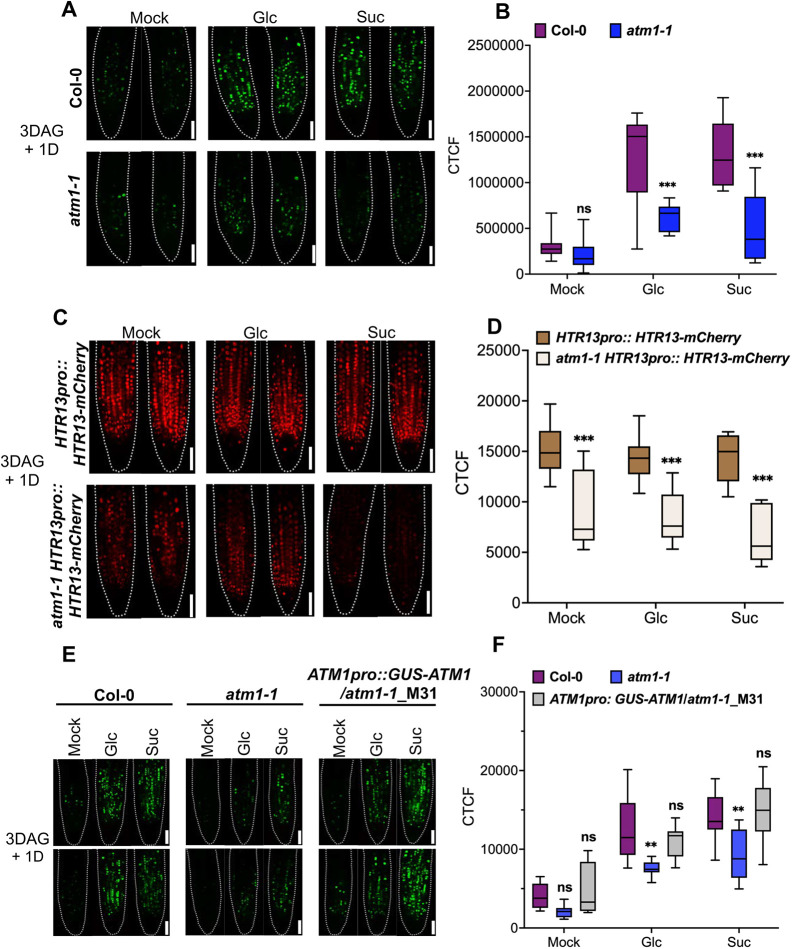
***atm1-1* is defective in S-phase entry of the cell cycle.** (A) EdU staining reveals a reduced number of proliferating cells in *atm1-1* root meristems. Quiescent 3-day-old Col-0 and *atm1-1* seedlings were treated with 0.5× MS (mock) or supplemented with either 15 mM glucose (Glc) or sucrose (Suc) and grown for an additional day at 15 µmol m^−1^ s^−1^ 12 h light/12 h dark conditions before EdU staining *in situ*. Dashed lines outline the root apical meristem. (B) Quantified average corrected total fluorescence (CTCF) in Col-0 and *atm1-1* under different treatment conditions. *N*=10; ns, not significant; ***P<0.001, two-way ANOVA and Tukey's multiple correction test. (C) The *HTR13pro::HTR13-mCherry* reporter from the PlaCCI line shows diminished expression in *atm1-1*. Dashed lines outline the root apical meristem. (D) Mean total mCherry fluorescence in plants expressing *HTR13pro::HTR13-mCherry*. *N*=9-10; ***P<0.001, two-way ANOVA and Tukey's multiple comparison test. (E) S-phase entry is restored in *ATM1pro::GUS-ATM1/atm1-1_M31* as shown by EdU staining *in situ*. Two representative images are shown in the top and bottom rows for each genotype and treatment. (F) CTCF in Col-0, *atm1-1* and *ATM1pro::GUS-ATM1/atm1-1_M31* under different treatment conditions. *N*=10; ns, not significant; ***P<0.01, two-way ANOVA and Tukey's multiple correction test. Box plots extend from 25th to 75th percentile; horizontal lines represent median; whiskers represent minimum to maximum values. Scale bars: 50 µm.

In addition to auxin marker genes being differentially expressed in *atm1-1*, many cell cycle genes were also induced in *atm1-1* in response to sucrose treatment (Fig. 5C), including B-type CYCLIN genes. To verify whether cell cycle regulation is influenced by ATM1, we deployed nucleosides analog 5-ethynyl-2′-deoxy uridine (EdU) to label newly replicating DNA in the root meristems. Nuclear EdU staining is an ideal protocol to obtain information on S-phase cells within developing tissues (Echevarría et al., 2021). For effective investigation of cell cycle progression in *atm1-1* roots, we used 3-day-old mitotic quiescent seedlings grown under photosynthesis-constrained light conditions. These quiescent seedlings were treated with MS medium or with either glucose or sucrose for 24 h before *in situ* labeling of the roots with EdU. Under mock treatment, the EdU-labeled DNA in *atm1-1* was not significantly different from WT (Fig. 7A,B). Upon sugar exposure, the quantity of stained cells entry the S phase of cell cycle was significantly reduced in *atm1-1* compared with WT (Fig. 7A,B).

To further substantiate the role of ATM1 in control of cell cycle events, we crossed the previously described Plant Cell Cycle Indicator (PlaCCI) marker (Desvoyes et al., 2020) into *atm1-1*. The PlaCCI transgene contains three fluorescent markers to monitor cell cycle status, including *HTR13pro::HTR13-mCherry* that reveals cells in S or G2phase of the cell cycle. Compared with WT plants carrying *HTR13pro::HTR13-mCherry*, the expression of the HTR13-mCherry signal was significantly reduced in *atm1-1* RAM cells (Fig. 7C,D). We further examined the root meristem of the complemented lines to determine whether the competence of the *atm1-1* roots to undergo DNA replication is restored. Our results showed that the root meristem of the quiescent *ATM1pro: GUS-ATM1*/*atm1-1* seedlings labeled with EdU stain acquired the capacity to activate S-phase dynamics of the cycle when compared with WT plants root meristem (Fig. 7E,F). Taken together, these results suggest ATM1 plays a role in cell proliferation in the root meristem.

### TOR and HXK1 pathways are genetically linked to ATM1

**Fig. 8.**
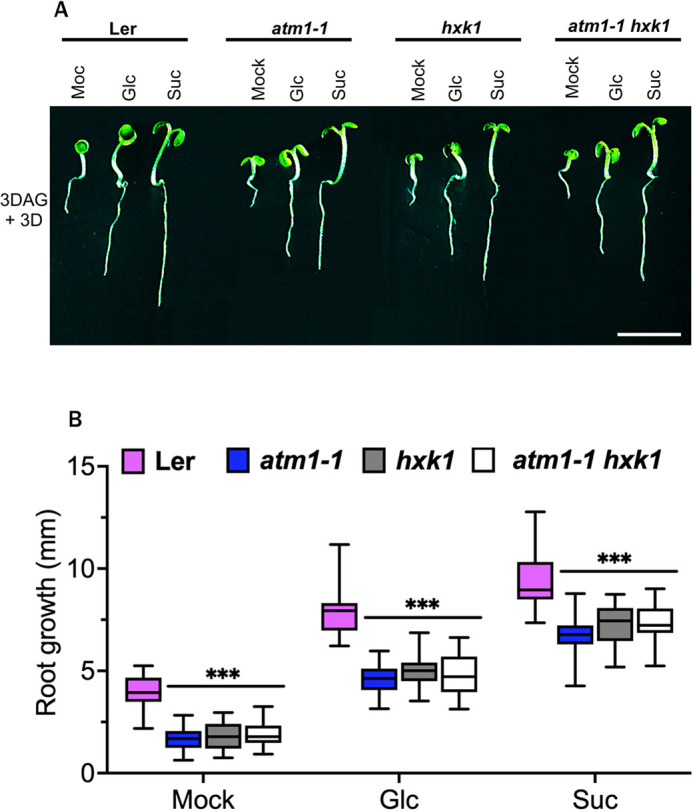
**Genetic analysis between *atm1-1* and *hxk1* indicates an epistatic interaction*.*** (A) Re-activation of root growth in 6-day-old wild-type (Ler), *atm1-1*, *hxk1-1* and *atm1-1 hxk1-1* seedlings in response to 0.5× MS medium (Mock) or medium supplemented with 15 mM glucose (Glc) or 15 mM sucrose (Suc) under 15 µmol m^−1^ s^−1^ 12 h light/12 h dark conditions. (B) Quantification of root growth across 3 days following Glc or Suc treatment in Ler, *atm1-1*, *hxk1-1* and *atm1-1 hxk1-1* seedlings. *N*=20; ***P<0.001, two-way ANOVA and Tukey's multiple comparison test. Scale bar: 1 mm.

Numerous sugars are known to control gene expression and cell proliferation in Arabidopsis, including sucrose, glucose, and trehalose-6-phosphate (Choudhary et al., 2022; Li et al., 2019; Wu et al., 2019; Xiong et al., 2013). Multiple pathways have been identified for sugar and energy sensing and signaling in plants, including TORC, HXK1 and SnRK1 (Artins and Caldana, 2022; Li and Sheen, 2016; Rolland et al., 2002; Smeekens and Hellmann, 2014). Given the defects in both glucose- and sucrose-induced root growth in *atm1-1* roots (Fig. 1), we wanted to determine whether any of these sugar-sensing pathways were impaired in *atm1-1*. In response to glucose, SnRK was inactivated via KIN10/11. In *atm1-1* seedlings, *KIN11* was repressed in response to glucose (Table S1), suggesting that SnRK signaling is normal in the mutant. We therefore focused on TOR and HXK1 pathways for genetic analyses with *atm1-1*. We crossed the previously described *hxk1* mutant (Moore et al., 2003) into *atm1-1* to generate the *hxk1 atm1-1* double mutant. Because glucose and sucrose are important for re-activation of cell proliferation in the meristem, we analyzed the response of 3-day-old mitotic quiescent seedlings of *hxk1 atm1-1* to both sugars. WT root growth is re-activated in response to both glucose and sucrose, which is impaired in both *hxk1* and *atm1-1* (Fig. 8A,B). The *atm1-1 hxk1* double mutant is the same as both parental phenotypes (Fig. 8A,B). This result indicates that ATM1 and HXK1 are in the same pathway.

**Fig. 9.**
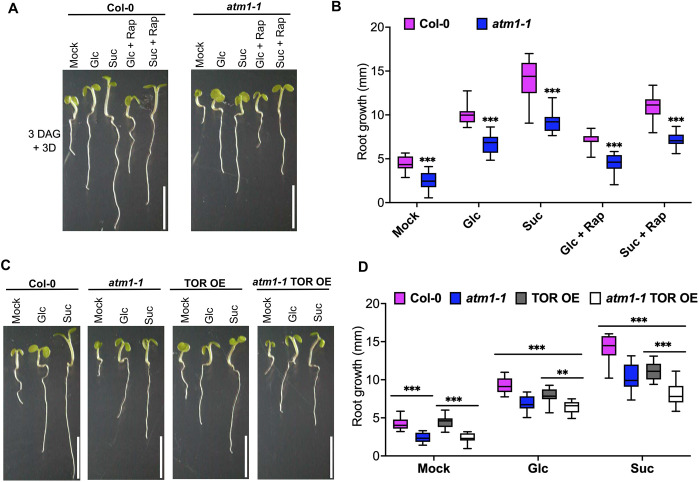
**Genetic interactions between TOR and ATM1.** (A) Inhibition of root growth re-activation in 6-day-old wild-type and *atm1-1* seedlings in response to 0.5× MS (mock) with or without 15 mM glucose (Glc) or sucrose (Suc) and in the presence of 10 µM rapamycin (Rap) under 15 µmol m^−1^ s^−1^ 12 h light/12 h dark conditions. (B) Quantification of root growth across 3 days following Glc, Suc and/or Rap treatment in Col-0 and *atm1-1* seedlings. *N*=16-18; ***P<0.001, two-way ANOVA and Tukey's multiple correction test. (C) Phenotypes of 6-day-old wild-type, *atm1-1*, TOR overexpression (TOR OE) and *atm1-1* TOR OE seedlings in response to 0.5× MS (mock) with or without 15 mM Glc or Suc under 15 µmol m^−1^ s^−1^ 12 h light/12 h dark conditions. (D) Evaluation of root growth in seedlings grown on 0.5× MS with/without Glc or Suc. *N*=16-18; **P<0.01, ***P<0.001, two-way ANOVA and Tukey's multiple correction test. Box plots extend from 25th to 75th percentile; horizontal lines represent median; whiskers represent minimum to maximum values. Scale bars: 5 mm.

Sugars are positive regulators of TOR kinase activity, and the role of glucose-TOR signaling has been implicated in transcriptional regulation of thousands of genes involved in nutrient/metabolic transport, cell cycle and anabolic processes (Dong et al., 2015; Xiong et al., 2013). Furthermore, blocking of TORC1 activity with chemical inhibitors has accelerated our understanding of the role of TOR kinase as a central integrator of phytohormones, sugars, nutrients and light signaling in plants (Cai et al., 2017; Li et al., 2017; Wu et al., 2019). To address the question of whether the TOR pathway is involved in the *atm1-1* root cell division defects, we investigated the ability of 3-day-old mitotic quiescent seedlings to restore root growth in the presence of sugars and a TOR kinase inhibitor, rapamycin. Under mock conditions, WT plants had significantly increased root growth compared with *atm1-1* (Fig. 9). In addition, root growth on glucose and sucrose was impaired in *atm1-1* compared with WT (Fig. 9A,B). In the presence of 10 µM rapamycin and sugar (either glucose or sucrose), *atm1-1* had diminished root development compared with WT and untreated *atm1-1* seedings (Fig. 9A,B). Thus, TOR activity can still be inhibited in *atm1-1* roots as assayed by rapamycin treatment. Next, we asked whether overexpression of TOR kinase in *atm1-1* background would be sufficient to restore the mutant short root growth. However, constitutive expression of TOR could not restore the stunted *atm1-1* root growth (Fig. 9C,D). Collectively, these results suggest that ATM1 is downstream of TOR signaling.

## DISCUSSION

In recent years, tremendous progress has been made in plant-specific myosin research, particularly in the area of auxin and sugar-regulated developmental processes (Abu-Abied et al., 2018; Han et al., 2021preprint; Holweg, 2007; Holweg et al., 2003; Ojangu et al., 2018; Olatunji and Kelley, 2020). Spatiotemporal studies on myosin expression patterns have revealed the accumulation of myosin XI and VIII in numerous tissues such as flowers, inflorescences, stems, siliques and roots, suggesting that myosins are required for development at the whole plant level (Haraguchi et al., 2014; Park and Nebenführ, 2013). The use of the *Arabidopsis* root system has extensively aided our knowledge of the molecular and cellular mechanism controlling root development from initiation and patterning to emergence, wherein many key and previously unreported gene functions have been identified (Casimiro et al., 2003; Celenza et al., 1995; Péret et al., 2009; Torres-Martínez et al., 2022). In this study, we investigated how an auxin-regulated class VIII myosin, ATM1, controls plant root development. Here, a GFP transcriptional reporter indicates that *ATM1* is expressed in primary roots (Fig. 1A) as previously reported (Clark et al., 2019; Haraguchi et al., 2014) and in secondary roots (Fig. 1B,C), indicating that *ATM1* may play a role in root development. Furthermore, *ATM1* expression occurs in a gradient along the RAM that is reminiscent of the PLETHORA genes, which are known to positively regulate RAM renewal in response to auxin cues (Mähönen et al., 2014; Santuari et al., 2016). However, ATM1 is not known to be a target of PLT transcription factors (Santuari et al., 2016). Other transcription factor(s) may be important regulators of *ATM1* expression in the RAM, which may be determined in future studies.

Myosin proteins are known to have complex subcellular localization patterns that can include cytoskeletal associations with actin and/or microtubules, plasmodesmal and/or plasma membrane localizations, or at newly formed cell plate (Golomb et al., 2008; Kurth et al., 2017; Nebenführ and Dixit, 2018; Peremyslov et al., 2010; Ryan and Nebenführ, 2018). Myosins can also be associated with vesicular trafficking and move cargo within the cell (Perico and Sparkes, 2018; Ryan and Nebenführ, 2018; Titus, 2018). Here, we confirm that ATM1 is a plasma-localized protein *in vivo*, with strong accumulation in the stem cell niche of the RAM (Fig. 2A). In addition, we also confirmed that ATM1 is a PD-localized protein (Fig. S2) as previously reported (Haraguchi et al., 2014). These data suggest that ATM1 may function at the membrane/PD to influence cellular properties within developing roots. Notably, the ortholog of ATM1 in *P. patens*, Myosin VIII, has been shown to influence developmental transitions and phragmoplast guidance during cell division (Wu and Benzanilla, 2014; Wu et al., 2011). Thus, our work and previous studies together suggest that ATM1/Myosin VIII have a conserved role in positively regulating cell division properties *in planta*.

Root morphogenesis is generally activated by sugar molecules (Li et al., 2017; Xiong et al., 2013) generated in the shoot via photosynthesis. To investigate links between photosynthetically derived sugars and the *atm1-1* short root phenotype, we used previously described low light conditions (Li et al., 2019). Under depleting endogenous sugar conditions, the short RAM phenotype in *atm1-1* could be attributed to reduced uptake of exogenously applied sugar by the roots (Fig. 2B-E). The elevated GUS activity in *ATM1pro:GUS-ATM1* in the shoot apex, a region of active cell division, suggests that ATM1 plays a key role in regulation of cell proliferation in response to endogenous cues (Fig. S1A-D). The increased GUS-ATM1 activity in the shoot apex could be linked in part to sugar accumulation, as previous studies have shown that glucose and light control cell division at the shoot tip (Li et al., 2017). The use of non-metabolizable analogs of glucose and sucrose have been widely used to understand the role of sugar-mediated signaling pathway in plants (Cortès et al., 2003; Fernie et al., 2001; Gonzali et al., 2005). In addition, turanose insensitivity is associated with altered auxin homeostasis and altered expression of *WOX5*, a central organizer of the QC (Gonzali et al., 2005). To investigate the response of *atm1-1* roots to non-metabolizable sugars, the mutant and WT Col-0 were exposed to turanose, palatinose and 3-OMG. None of these non-metabolizable sugars significantly reactivated *atm1-1* root growth compared with WT. However, we cannot rule out the fact that the non-metabolizable sucrose isomer palatinose may act as a signaling molecule (Ramon et al., 2008), as it slightly stimulated shoot and root growth in *atm1-1* (Fig. 2F).

Moreover, the fact that *atm1-1* showed impaired root growth under normal photosynthetic conditions (Fig. 3C,D) corroborates the idea that the short root phenotype is due to defects in shoot-to-root sugar transport. In addition, the *atm1-1* short root phenotype can be complemented by a *ATM1pro::GFP-ATM1* transgene (Fig. 3B,D), which opens the door for future biochemical and imaging studies with this fluorescently tagged version of ATM1 expressed under a native promoter.

Studies have shown that altered sugar metabolism can cause a delay in distal stem cell differentiation (Pignocchi et al., 2021; Wang et al., 2018). Furthermore, exogenous application of sugars has been reported to restore delayed distal stem cell in several mutants, suggesting that there is a common regulatory pathway underpinning this process (Pignocchi et al., 2021; Racolta et al., 2014; Wang et al., 2018). Therefore, the diminished expression of the columella cell marker *PET111:GFP* in *atm1-1* grown in the presence of sucrose indicates that ATM1 modulates columella stem cell initial formation via a sugar-dependent pathway (Fig. 4C,D). In addition, we showed that sugar treatment (glucose or sucrose) completely reinstates the number of differentiated columella stem cells in *atm1-1* mutant, but not their cell size (Fig. 4E). Based on these findings, we propose that ATM1 plays an essential role in the proper division and maintenance of columella stem cell initials via a sugar-mediated pathway.

The interplay between sugar and auxin has been shown to influence diverse aspects of plant development including root architecture and growth (Mishra et al., 2009, 2022). Previous work has demonstrated that exogenous application of sugars promotes auxin biosynthesis and auxin-regulated gene expression (Mishra et al., 2009, 2022; Sairanen et al., 2012) Here, the examination of glucose- and sucrose-regulated gene expression in WT and *atm1-1* seedlings revealed the downregulation of genes involved in auxin signaling, transport and catabolism in the presence of exogenously applied sugars (Fig. 5B,C). These expression data suggest that ATM1 is required for auxin homeostasis in seedlings, which is well-known to be required for RAM size and function. The diminished expression pattern of *DR5:GFP* in *atm1* (Fig. 6A,B) mimics that of auxin biosynthesis mutants with impaired auxin response (Brumos et al., 2018; Stepanova et al., 2008), indicating that ATM1 may be linked to auxin pathways in a sugar-dependent fashion because *DR5:GFP* was increased in *atm1-1* upon sugar supplementation. Although class XI myosins have been implicated in auxin responses, this is the first report linking a class VIII myosin to auxin-mediated root development (Abu-Abied et al., 2018; Han et al., 2021 preprint; Holweg, 2007; Holweg et al., 2003; Ojangu et al., 2018).

Previous studies have shown that cell proliferation in the root meristem is controlled by cell cycle events governed by transcription factors (Gutierrez, 2022; Li et al., 2017; Ornelas-Ayala et al., 2020; Sozzani et al., 2010; Xiong et al., 2013). Cell cycle progression from one phase to another is a tightly regulated process and requires mitogenic signals mainly at the G1/S and G2/M transitions (Desvoyes et al., 2020; Gutierrez, 2022). Sugars are potent mitogenic signals that mediate the progression of cell cycle from G1 to S phase (Li et al., 2017; Xiong et al., 2013). Here, using EdU staining, we provide previously unreported evidence on the role of ATM1 in the activation of S phase of the cell cycle during meristem formation in response to sugar cues (Fig. 7A,B). Similarly, we showed that the expression of H3.1-mCherry (an early S-phase marker in the PlaCCI line previously described by Desvoyes et al. (2020) was altered in *atm1-1* root meristems, indicating that ATM1 is a positive regulator of cell cycle progression in the RAM (Fig. 7C,D). In addition, we have observed that two B-type and one P-type CYCLIN (CYC) genes are elevated in *atm1-1* in response to sucrose treatment: *CYCB2;4*, *CYCB1;4*, *CYCB2;3*. B-type cyclins control microtubule organizations during cell division in *Arabidopsis*, with *CYCB1;2* having established roles in root cytoskeletal regulation (Romeiro Motta et al., 2022). Furthermore, a drop in B-type CYCLIN expression levels is associated with promotion of cell cycle progression. Thus, *atm1-1* roots appear to have impaired sucrose-induced cell cycle progression as a result of persistent cyclin expression. Glucose-mediated activation of TOR kinase and subsequent phosphorylation of E2Fa (a cell cycle transcriptional activator) has been implicated in root meristem maintenance (Li et al., 2017). The inability of exogenously applied glucose to rescue the root meristem activity inhibited by rapamycin (a TOR Kinase potent inhibitor) or in *tor* mutant provides a link for TOR in sugar-mediated RAM development (Xiong et al., 2013). The additive effect of inactivation of TOR with rapamycin in *atm1* mutant may indicate a possible role of ATM1 in TOR-mediated root meristem maintenance, but this would require further research. Recently, TORC1 was shown to control energy levels to regulate actin cytoskeleton in *Arabidopsis* (Dai et al., 2022), providing previously unreported insights on how plants control energy situations.

**Fig. 10.**
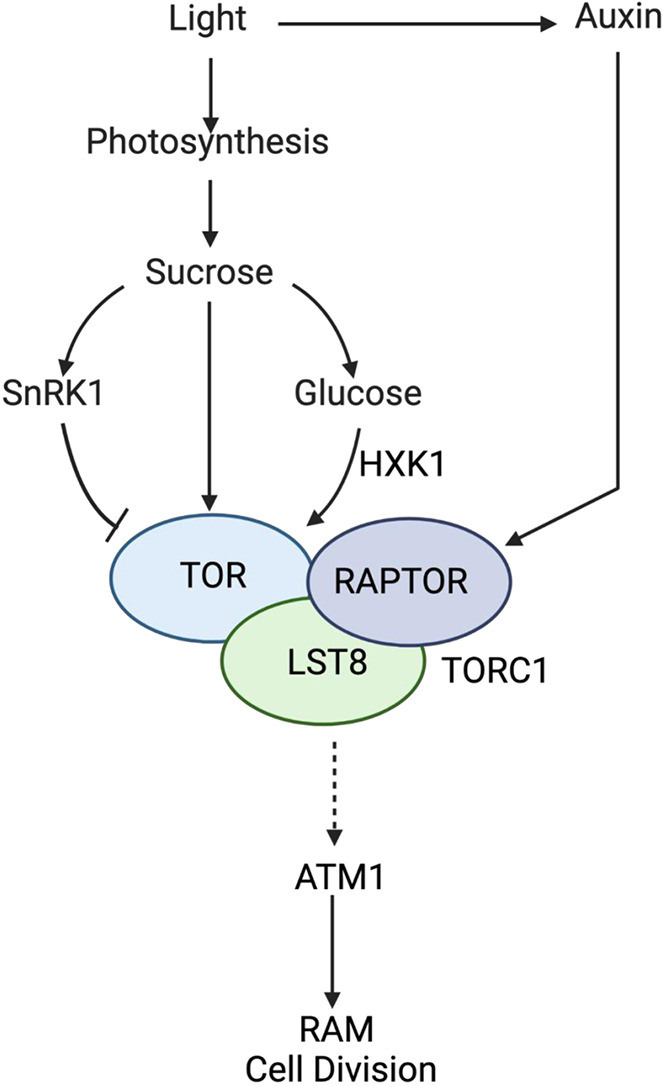
**Proposed framework for ATM1 function.** Under photosynthetic conditions, two shoot-derived growth cues, sucrose and auxin, are integrated by TORC1, which includes partner proteins RAPTOR and LST8. The glucose sensor HXK1 and sucrose sensor SnRK1 are also linked to sugar-sensing and TORC1 activity. ATM1 is downstream of these sugar-sensing pathways and is required for proper cell proliferation in the root. The dashed line represents an unknown molecular mechanism and/or factors in between sugar sensors and ATM1.

Together, these results lead us to propose a working model (Fig. 10) whereby ATM1 positively influences RAM cell proliferation in a sucrose-dependent manner, downstream of TOR, which acts as part of a complex with RAPTOR and LST8. ATM1 is also likely downstream of HXK1, given the observed glucose defects in *atm1-1* roots and the genetic interactions between ATM1 and HXK1. Both of these developmental processes occur in response to two key growth cues, sugars and auxin. Loss of *ATM1* impairs root morphogenesis under low-light or low-sugar growth conditions, suggesting that sugar signaling may be linked to the activity of this particular myosin. In conclusion, our findings on the role of ATM1 in modulating root meristem cell cycle state and stem cell differentiation provide a missing link on the role of plant-specific class VIII myosins in plant growth and development.

## MATERIALS AND METHODS

### Plant materials and growth conditions

All *Arabidopsis thaliana* plants used in this work were in the Columbia (Col-0) accession background except for *gin2-1*, an allele of HEXOKINASE 1, which is in Landsberg (Ler) background. T-DNA insertion mutant SAIL_405_B08 (*atm1-1*) was obtained from the *Arabidopsis* Biological Resource Center (ABRC; https://abrc.osu.edu/) and homozygous lines were identified by PCR using gene-specific primers (Table S2); this null allele has been described previously (Olatunji and Kelley, 2020). The fluorescent reporter lines used in this study were crossed into *atm1-1*, carried through the F3 generation and verified by PCR-based genotyping at each generation. This includes *WOX5:GFP* (Brady et al., 2007), *PET111:GFP* (Brady et al., 2007), TOR OE (Pu et al., 2017), *DR5:GFP* (Friml et al., 2003) and the PlaCCI line (Desvoyes et al., 2020).

Before planting, seeds were surfaced sterilized using 50% bleach and 0.01% Triton X-100 for 10 min and then washed five times with sterile water. Seeds were then imbibed in sterile water for 2 days at 4°C. The growth conditions used for experiments were 12 h white light/12 h dark at 22°C. The light intensity was 45 μmol m^−1^ s^−1^ except where otherwise stated.

For sugar-induced reactivation of root growth assays, sterilized seeds were incubated in sugar-free liquid medium 0.5× MS without vitamins (MSP01, Caisson), pH 5.7, for 2 days at 4°C, and then germinated in low light (15 μmol m^−1^ s^−1^, 12 h light/12 h dark, 22°C) for 3 days to enter a mitotically quiescent state as described previously (Li et al., 2019). The quiescent seedlings were then transferred to 0.5× MS medium supplemented with 15 mM glucose or 15 mM sucrose and incubated for three additional days in weak light (15 μmol m^−1^ s^−1^, 12 h light/12 h dark, 22°C) before root meristem and root growth analysis.

All primers used for cloning are listed in Table S2. To generate *ATM1pro::GFP-ATM1* and *ATM1pro::GUS-ATM1* constructs, a 4.5 kb genomic region upstream of the first annotated ATG in the *ATM1* gene was amplified using gene-specific primers and ligated into pENTR5′ (Addgene plasmid #27320) to create plasmid pDO#22 [pENTR5′-ATM1pro (4.5 kb)]. The GFP sequence without the stop codon was amplified from pEGAD/CD3-389 (Cutler et al., 2000) and cloned into the pDONR221 entry vector to create pDO#06 (L1-EGFP-L2). The GUS coding sequence was amplified from pMDC163 and cloned into entry clones pDONOR 221 and pDONOR P2R-P3 to create pDO#48 (L1-GUS-L2) and pDO#49 (R2-GUS-L3), respectively. To create the *ATM1pro::NLS-GFP-GUS* (pDO#86) construct, pEN-L1-NF-L2 (entry vector containing NLS-GFP sequences was obtained from VIB Ghent University) and pDO#49 (R2-GUS-L3) were recombined into pK7m34GW via LR Clonase reaction. A full-length *ATM1* genomic fragment (6950 bp) was amplified from the first start codon to the stop codon using gene-specific primers and inserted into pDONOR P2R-P3 entry vector via a BP Clonase reaction to create pDO#25 (R2-ATM1 FL-L3). The final constructs were created by cloning the entry clones into pK7m34GW destination vector using Multisite Gateway system to generate the expression clones: pDO#28 (*ATM1pro:GFP-ATM1*) and pDO#44 (*ATM1pro:GUS-ATM1*) which were individually transformed into chemically competent *Agrobacterium tumefaciens* strain GV3101 and used for the transformation of *Arabidopsis* plants by the floral dip method as previously described (Clough and Bent, 1998).

### Transient expression and plasmodesmal callose staining in *Nicotiana benthimiana*

For *A. tumefaciens*-mediated transient expression, overnight cultures of *A. tumefaciens* strain GV3101 harboring *ATM1pro: GFP-ATM1* (pDO#28) plasmid were centrifuged and adjusted to a desired bacterial density of 2×10^−1^ (A600) using sterilized water, and then infiltrated into the abaxial side into the fourth leaves of 5-week-old *N. benthamiana* leaves. Callose deposits at the PD were observed with Aniline Blue (0.01% in 1×PBS buffer, pH 7.4) 48 h post infiltration. Plasmodesmal deposits into the leaves were visualized 15 min after dye infiltration with the confocal microscope.

### EdU staining

EdU staining was carried out as previously described (Xiong et al., 2013). Briefly, surface sterilized seeds were imbibed in sugar-free liquid medium [0.5× MS without vitamins (pH 5.7)], for 2 days at 4°C and subsequently grown under weak light intensity (15 μmol m^−1^ s^−1^, 12 h light/12 h dark, 22°C) for 3 days in a Percival growth chamber to enter a mitotically quiescent state. The quiescent seedlings were transferred into 0.5× MS medium supplemented with 15 mM glucose or 15 mM sucrose for 1 day before EdU staining. Seedlings were stained with 1 μM EdU for 30 min and then fixed for 30 min in 3.7% formaldehyde solution in PBS solution with 0.1% Triton X-100. After the fixative removal, the seedlings were washed three times for 10 min with PBS solution. The washed seedlings were then incubated in EdU detection cocktail (Invitrogen) for 30 min at room temperature in the dark, followed by PBS solution washing three times (10 min each). The root meristems of fixed seedlings were observed using a Zeiss LSM 700 confocal microscope.

### Lugol staining

Five-day old seedlings germinated on 0.5× MS plates containing 15 mM glucose or 15 mM sucrose were incubated in a root cap fixative solution [5% (w/v) formaldehyde, 5% (v/v) acetic acid, and 25% (v/v) ethanol] for 24 h and then briefly stained with Lugol solution for 30 s. Fixed and stained roots were mounted onto microscopic slides and cleared with chloral hydrate:glycerol:water (8:3:1 ratio) before imaging with the Thunder Imaging Systems Microscope (Leica).

### Histological GUS staining

Plant tissues were initially fixed in 90% acetone at −20°C for 30 min. Samples were stained with GUS staining solution [0.5 mM ferrocyanide, 0.5 mM ferricyanide, 25 mg 5-bromo-4-chloro-3-indotyl β-D-galactopyranoside sodium salt (X-Glc; Sigma-Aldrich) dissolved in 0.5% (v/v) dimethylformamide (DMF), 500 mM potassium-phosphate buffer (pH 7.2) buffer, 0.5 mM EDTA (pH 8) and 0.5% (v/v) Triton X-100], vacuumed for 10-40 min, followed by incubation in the dark at 37°C. Staining solution was washed off by rinsing tissue in potassium phosphate buffer. Tissues were fixed in 3:1 ethanol/acetic acid for 3 h, thereafter cleared in a 50% ethanol and in chloral hydrate:water:glycerol (8:2:1, w/v/v) for 24 h before imaging using a Leica M165 FC fluorescence stereomicroscope equipped with a DMC4500 digital camera.

### Transcriptomic and GO enrichment analyses

Total RNA was extracted from 5-day old Col-0 and *atm1-1* seedlings grown on 0.5× MS media supplemented with 15 mM glucose, 15 mM sucrose or no sugar (‘mock’); three biological replicates per genotype and treatment were collected. Total RNA was extracted using Trizol reagent followed by column clean up with a Zymo Direct-zol RNA purification kit. RNA quality was measured using a Bioanalyzer. Total RNA concentrations were determined using a NanoDrop and Qubit. QuantSeq 3′ mRNA libraries were prepared using the Lexogen 3′ mRNA-seq FWD kit and sequenced on an Illumina HiSeq 3000 as 50 bp reads. QuantSeq reads were mapped to the TAIR10 genome and differential gene expression analysis was performed using PoissonSeq implemented in R (Li et al., 2019). Raw QuantSeq data are deposited at GEO with accession GSE200917. Transcripts with a *q*-value ≤0.1 were assigned as differentially expressed. GO enrichment analysis was performed in PANTHER using the *A. thaliana* reference genome with a Fisher's Exact test type and a false discovery rate correction and GO terms with corrected *P*-value less than 0.05 were considered significantly enriched. GO terms enrichment for biological process was conducted using REVIGO (Supek et al., 2011) with a stringent dispensability cut-off (*P*-value<0.05) and were plotted with an R script (Bonnot et al., 2019).

### RT-PCR

All primers used for PCR assays are listed in Table S2. Total RNA extracted from 5-day-old whole seedlings was purified as described above and 1 µg of total RNA was used for cDNA synthesis using the LunaScript RT SuperMix Kit (New England Biolabs) according to the manufacturer's instruction. For RT-PCR, gene-specific primers were used to amplify the target sequences for 35 cycles. PCR products were separated on 2% agarose gel.

### Confocal microscopy

Zeiss laser scanning microscope (LSM) 700 was used for confocal imaging. For confocal imaging of the RAM, roots were stained with either propidium iodide (PI) or EdU. Fluorescent signals were excited with the following laser lines: GFP (488 nm) and PI (555 nm). The signals were then collected with the following emissions: GFP (555 nm) and PI (640 nm). Zeiss LSM 780 was used for imaging of *HTR3pro::HTR3-mCherry* expression from the PlaCCI transgene. The mCherry fluorescence excitation and emission used in this study was 610 nm and 580-610 nm, respectively.

### Statistical analysis

GraphPad Prism Software, version 9.3.1 was used for statistical analysis, using two-way ANOVA and Tukey's multiple comparison test. The experiments were performed at least in duplicates, but only the data from one representative experiment is shown.

## Supplementary Material

10.1242/develop.201762_sup1 Supplementary information
