# Supplementary material for: The class VIII myosin ATM1 is required for root apical meristem function
Source: Development. 2023 Jul 4;150(20):dev201762. doi: 10.1242/dev.201762 (PMC10357013; doi:10.1242/dev.201762)
Supplement: Supplementary information [file develop-150-201762-s1.pdf]

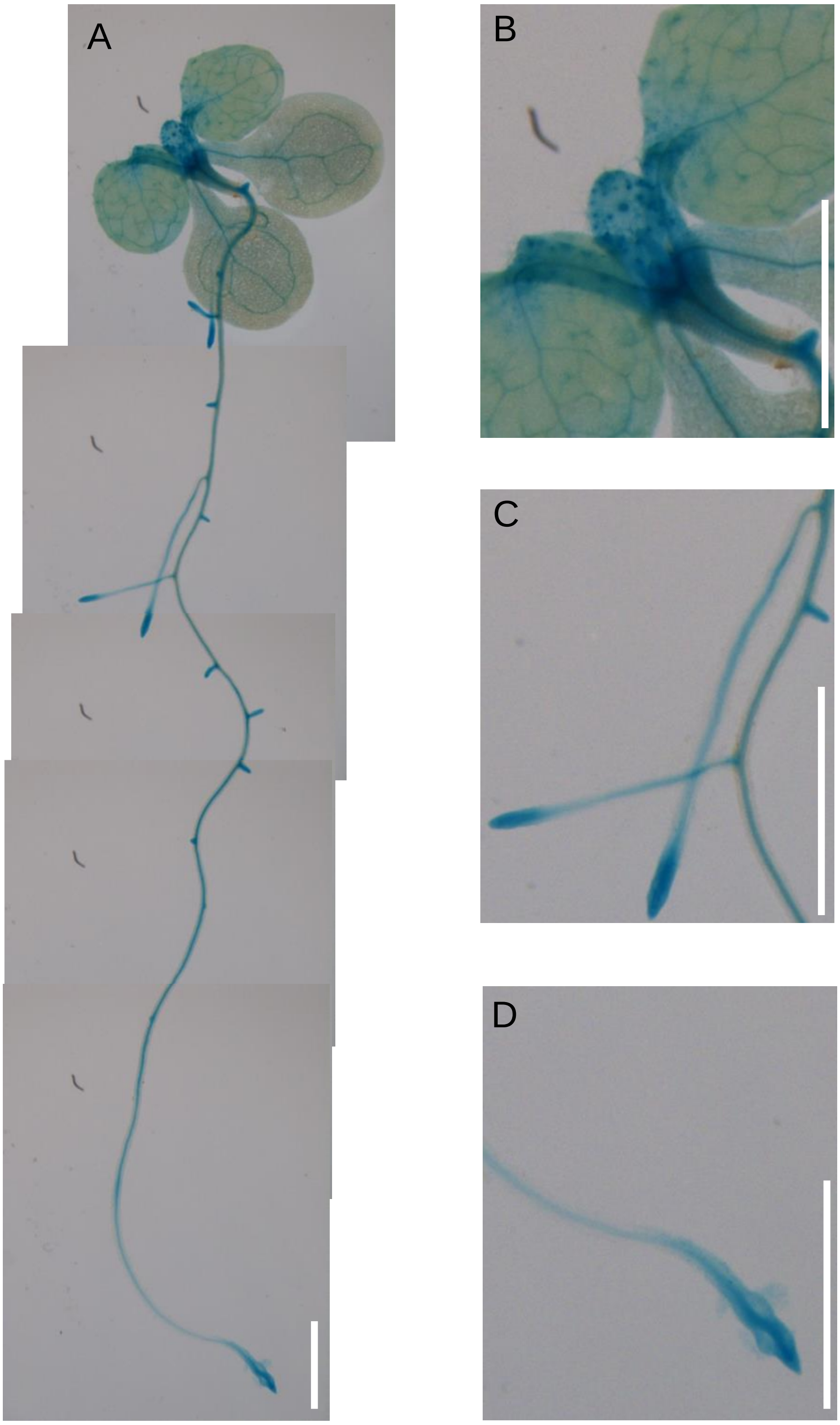

Fig. S1. Histological staining of GUS activity in Arabidopsis seedlings expressing *ATM1pro::GUS-ATM1*. (A) Tissue-specific expression of GUS-ATM1 in a representative 6-day-old seedling. (B-d) Enlarged images of GUS-ATM1 activity in the shoot apical meristem (B), lateral roots (C), and the primary root (D). Scale bars = 2.8 mm.

Development • Supplementary information

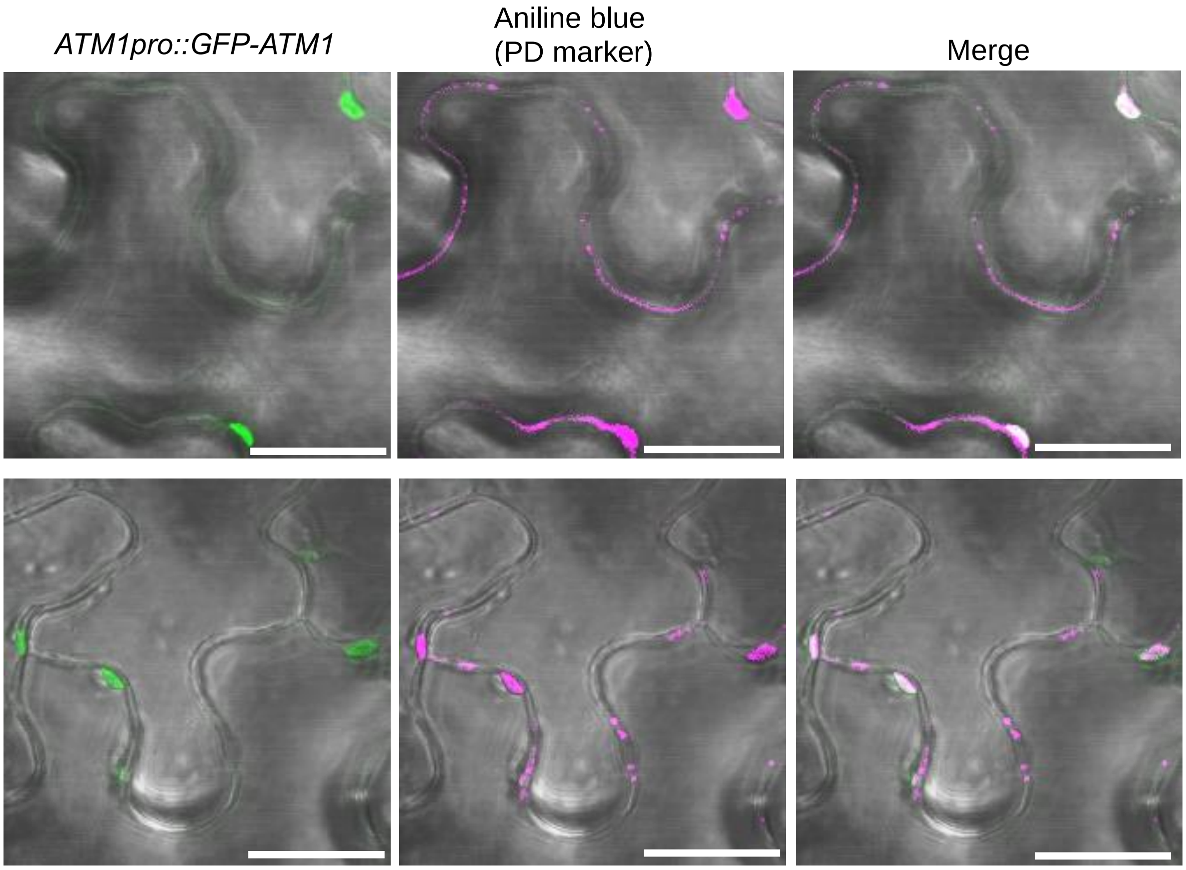

Fig. S2. Co-localization of ATM1 with a plasmodesmata marker. *Nicotiana benthimiana* leaves transiently expressing *ATM1pro::GFP-ATM1* were infiltrated with aniline blue fluorochrome solution to stain for plasmodesmata (PD). Representative images were taken 48 h post agroinfiltration using a confocal microscope. White arrows depict punctate accumulation of GFP-ATM1 between adjacent epidermal cells, coincident with PD staining. Aniline blue was false colored magenta for contrast with GFP signal. Scale bars = 20 µm.

Development • Supplementary information

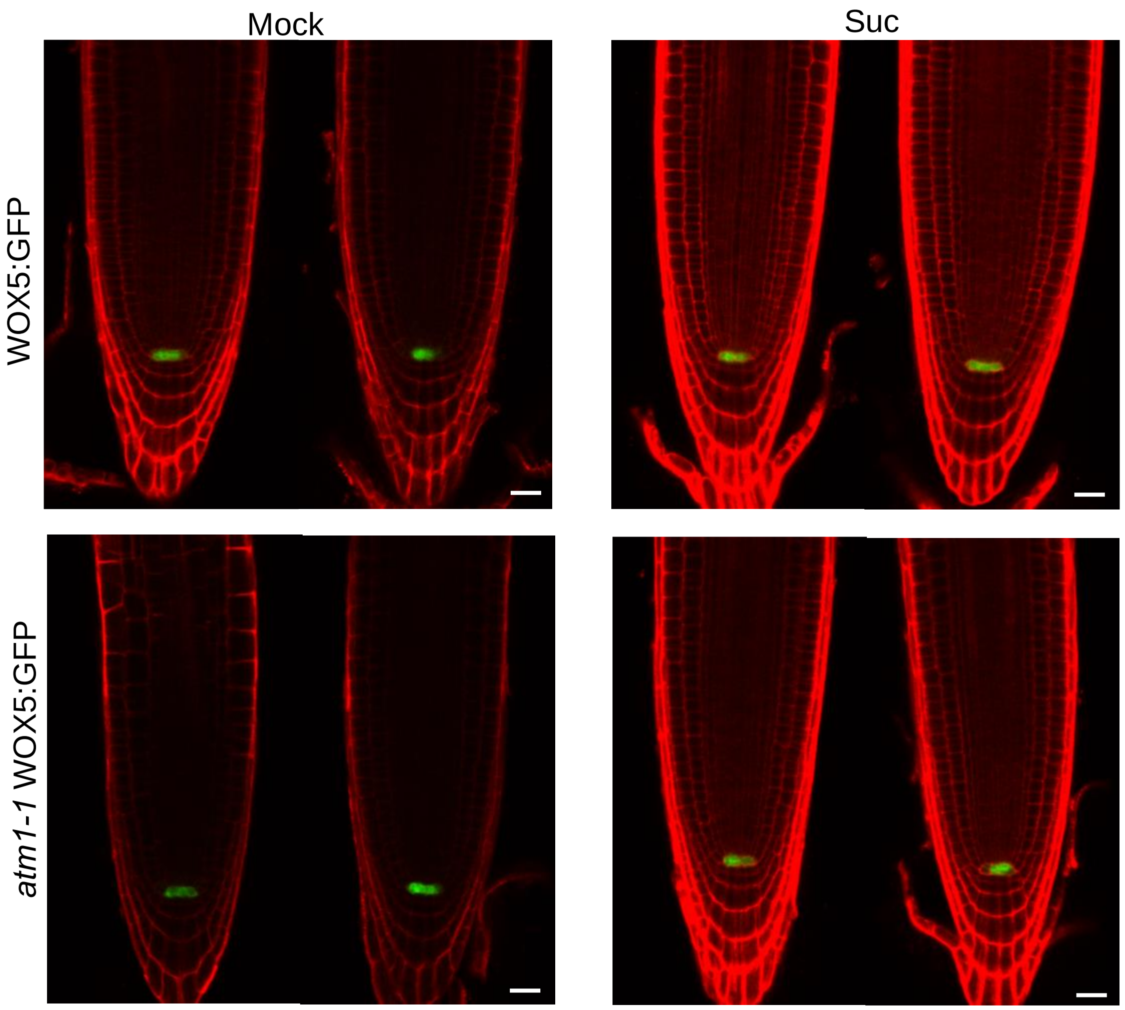

Fig. S3. The expression of the quiescent center marker *WOX5:GFP* is normal in *atm1-1* root meristems. The roots of 5-day-old seedlings grown on 0.5X MS medium (mock) or supplemented with 15 mM Sucrose were counterstained with propidium iodide prior to imaging. Scale bars = 20 µm

Table S1. TPM-normalized values from QuantSeq for each gene in each sample.

Click here to download Table S1

Table S2. Primers used in this study for genotyping, cloning, and RT-PCR.

Click here to download Table S2
