# Supplementary material for: The class VIII myosin ATM1 is required for root apical meristem function
Source: Development. 2023 Jul 4;150(20):dev201762. doi: 10.1242/dev.201762 (PMC10357013; doi:10.1242/dev.201762)
Supplement: Supplementary file 1 [file develop-150-201762_review_history.pdf]

The class VIII myosin ATM1 is required for root apical meristem function

Damilola Olatunji, Natalie M. Clark and Dior R. Kelley
DOI: 10.1242/dev.201762

Editor: Ykä Helariutta

Review timeline

Original submission:	11 October 2022
Editorial decision:	12 December 2022
Resubmission:	9 March 2023
Editorial decision:	11 April 2023
First revision received:	11 May 2023
Accepted:	30 May 2023

Original submission

First decision letter

MS ID#: DEVELOP/2022/201372

MS TITLE: The Class VIII myosin ATM1 is required for root apical meristem function

AUTHORS: Damilola Olatunji, Natalie M. Clark, and Dior Kelley

ARTICLE TYPE: Research Article

I have now received all the referees reports on the above manuscript, and have reached a decision. The referees' comments are appended below, or you can access them online: please go to BenchPress and click on the 'Manuscripts with Decisions' queue in the Author Area.

As you will see from their reports, the referees recognise the potential of your work, but they also raise significant concerns about it. Given the nature of these concerns, I am afraid I have little choice other than to reject the paper at this stage.

However, having evaluated the paper, I do recognise the potential importance of this work. I would therefore be prepared to consider as a new submission an extension of this study that contains new experiments, data and discussions and that address fully the major concerns of the referees. The work required goes beyond a standard revision of the paper. Please bear in mind that the referees (who may be different from the present reviewers) will assess the novelty of your work in the context of all previous publications, including those published between now and the time of resubmission.

Reviewer 1

Advance summary and potential significance to field

This paper examines the phenotypic consequences of a specific myosin XIII, ATM1. In general, individual myosins have not been rigorously examined, therefore careful phenotypic analysis is

© 2023. Published by The Company of Biologists under the terms of the Creative Commons Attribution License (https://creativecommons.org/licenses/by/4.0/).

essential in teasing apart the cellular and organismal functions of these essential proteins. The authors focus on root phenotypes, and find that this myosin is essential for differentiation of specific cell types and meristematic growth. Growth on sugars (sucrose and glucose) exacerbate the phenotype, and transcriptomic analysis suggest that auxin-driven processes are essential for the phenotypes observed.

Comments for the author

Major concerns
I am a bit ambivalent about my primary major concern, which is only a single allele was analyzed. The reason I am ambivalent is because molecular complementation was successfully performed, and from a previous study from the Kelley lab, it was shown that other readily available alleles are not nulls. None-the-less, I would feel much better about the study if some of the simpler phenotypes, such as root length in response to glucose and sucrose, were done with another allele.

My other concern is about how the data are discussed and the conclusions that are reached. Some discussion is a bit sparse, and I am not sure that a role for sugar signaling is explicitly shown here. My interpretation of the results is that glucose and sucrose both stimulate growth in wild type plants, and sucrose has a greater effect on stimulating meristem size and cell division. The atm1 mutant is less competent to respond. I think that the very nice experiment with non-hydrolyzable sugars shows that this is NOT (primarily) a signaling mechanism, but rather a metabolic one – which is no less interesting, just different. This might be semantics, but I think relevant in the context of hormones.

I'd like to see a more extensive discussion of the differences between the glucose and sucrose responses. The authors went to great expense to do transcriptomics with both sugars, but don't really explain why when setting up the experiment in the results, or in the discussion. "Previous studies have examined the effects of sucrose and glucose on transcription in Arabidopsis" – perhaps a sentence or two explaining why it that is relevant here. I'd also like see an attempt to connect (or not?) the cell differentiation phenotype with the growth phenotype. It is unclear to me whether the columella cell phenotype is connected at all with growth or auxin production, or just an unrelated effect.

Considering the discussion of auxin, I considered if additional experiments using exogenous auxin would help clarify the interpretation of results, but I think it would be more likely to convolute the data and are likely beyond the scope for this paper, and is unnecessary.

Minor concerns

In some places, class IX (nine) instead of XI (eleven) were written. A find & replace should easily fix this.

Regarding ATM1pro:NLS-GFP expression. It would be helpful to have a complementary supplemental data figure showing more confocal images and/or GUS images along the entire root length; it appears that ATM1 expression, based on Figure 1A and 2A likely extends beyond the meristematic region.

"Confocal microscopy of GFP-ATM1 in Arabidopsis roots revealed protein accumulation at the plasma membrane.." From the image shown, I don't think this can be concluded, but I also don't think it is essential for the conclusions in the paper to definitely show subcellular localization. I recommend "appears ATM is predominantly localized to the cell periphery". This could be cortical localization, and there is maybe some cytoplasmic localization? The pattern is consistent for myosins, thus I am not overly concerned.

Similarly, "Despite the mis-expression of the root cap markers in atm1-1, surprisingly, GFP expression of the QC marker WOX5:GFP is intact the mutant" - it is very hard to see this at the magnification shown. Can more magnified images, where the cells of the QC are clear, be shown? I think given the columella cell phenotype, this was a useful experiment, so clearer images are helpful.

© 2023. Published by The Company of Biologists under the terms of the Creative Commons Attribution License (https://creativecommons.org/licenses/by/4.0/).

Microscopy images should be color-blind friendly by replacing red with magenta. This can be done very quickly in FIJI using Image>color>replace red with magenta or in Photoshop by copy and pasting the red channel into the blue channel.

The first paragraph of the transcriptomic analysis reports the number of DEG for different comparisons – there are a lot of pairwise comparisons. This is all in the supplemental data, but it would be very helpful for the first tab in the data to have a table of the DEG between comparisons. Notably, there are relatively few genes different comparing atm1 and WT mock treated, while a more when treated with glc and even more with suc. I think this is helpful to note. The GO enrichment in 5D does not clearly convey this. I think the number of responding genes in the different genotypes/conditions is actually interesting as it (I think?) reflects the phenotypic responses – i.e. greater differences in both phenotype and gene expression are seen in the presence of sucrose, vs untreated.

A fair amount of work on myosin VIII had been done in moss, including work on development. Myosin VIII defects lead to a decrease in cell streaming and overall growth, which I think could be mechanistically similar. I think citing these studies is appropriate.

Wu, S. Z., Ritchie, J. A., Pan, A. H., Quatrano, R. S., & Bezanilla, M. (2011). Myosin VIII regulates protonemal patterning and developmental timing in the moss Physcomitrella patens. Molecular plant, 4(5), 909-921.

Wu, S. Z., & Bezanilla, M. (2014). Myosin VIII associates with microtubule ends and together with actin plays a role in guiding plant cell division. Elife, 3, e03498.

Reviewer 2

Advance summary and potential significance to field

The manuscript by Olatunji, Clark and Kelly, characterizes the expression domains of Arabidopsis thaliana Myosin1 (ATM1), a class XII-type myosin, in the root and lateral root meristem and shows that the atm1-1 mutant displays defects in sugar-dependent root and meristem length. The defects can be rescued by expression of GFP-ATM1 under control of its own promoter in the mutant background. The proposed plasma membrane (PM)localization of GFP-ATM1 is not addressed with appropriate markers or sufficient resolution here, but was parly indicated by earlier antibody studies at the electron microscopic level (Reichelt et al., 1999, Plant J.). The authors find the layer of columella stem cell daughter cells lacking in the atm1-1 mutant and interpret this as a defect in the maintenance of CSC identities, which, however is not strictly distinguishable from reduced cell division activity of the columnella stem cells (CSC) by the methods employed. The authors performed a transcriptomic analysis of wild type and the atm1-1 mutant in response to exogenously applied sucrose and glucose and founnd some auxin biosynthesis and response genes were upregulated in the atm1-1 mutant upon sucrose induction, while the auxin response reporter DR5:GFP displayed lower expression in atm1-1 than in the wild type. A mechanistic explanation for this is not provided, nor how this relates to myosin function. Analysis of c5-ethynyl-2-deoxyoridine (EdU) label to detect replicating cells in the root meristem and of a multi-cell-cyle-stage marker revealed a lower number of cells entering S and G2 phase in atm1-1 compared to the wild type. The findings would be of rather specific interest to some plant biologist, but do not convey a mechanistic understanding of ATM1 function in cell cycle progression and with respect to auxin responses and/or biosynthesis,

Comments for the author

Taken together, while the phenotypic characterization of the atm1-1 mutant is new and the experiments generally thoroughly performed, as far as they go, the study reveals little to no mechanistic insight into via which interactions ATM1 affects cell cycle progression auxin biosynthesis and/or response at the genetic and/or molecular level. This would need to be worked

© 2023. Published by The Company of Biologists under the terms of the Creative Commons Attribution License (https://creativecommons.org/licenses/by/4.0/).

out e.g. by genetic and/or molecular interaction studies in order to gain some mechanistic understanding of ATM1 function.

Author response to reviewers' comments

Reviewer 1

Comment 1.1: I am a bit ambivalent about my primary major concern, which is only a single allele was analyzed. The reason I am ambivalent is because molecular complementation was successfully performed, and from a previous study from the Kelley lab, it was shown that other readily available alleles are not nulls. None-the-less, I would feel much better about the study if some of the simpler phenotypes, such as root length in response to glucose and sucrose, were done with another allele.

Response 1.1: We understand the request for analysis of multiple alleles to address concerns regarding linking a gene to a function. Our group has previously published that the atm1-1 allele is a null while the other available T-DNA lines are not (Olatunji & Kelley 2020). As an alternative approach, we have generated multiple independent tagged lines of ATM1 (with GFP or GUS reporters) expressed under the endogenous (native) promoter which can complement the atm1-1 allele, restore root growth to normal, and rescue the observed cell division defects of atm1-1. Assaying root growth of other alleles that have wild-type levels of ATM1 activity would not be informative in this study.

Comment 1.2: My other concern is about how the data are discussed and the conclusions that are reached. Some discussion is a bit sparse, and I am not sure that a role for sugar signaling is explicitly shown here. My interpretation of the results is that glucose and sucrose both stimulate growth in wild type plants, and sucrose has a greater effect on stimulating meristem size and cell division. The atm1 mutant is less competent to respond. I think that the very nice experiment with non-hydrolyzable sugars shows that this is NOT (primarily) a signaling mechanism, but rather a metabolic one – which is no less interesting, just different. This might be semantics, but I think relevant in the context of hormones. I'd like to see a more extensive discussion of the differences between the glucose and sucrose responses. The authors went to great expense to do transcriptomics with both sugars, but don't really explain why when setting up the experiment in the results, or in the discussion. "Previous studies have examined the effects of sucrose and glucose on transcription in Arabidopsis" – perhaps a sentence or two explaining why it that is relevant here. I'd also like see an attempt to connect (or not?) the cell differentiation phenotype with the growth phenotype. It is unclear to me whether the columella cell phenotype is connected at all with growth or auxin production, or just an unrelated effect.

Response 1.2: We have added additional genetic analyses and extensive text edits to further elucidate the glucose versus sucrose dependent growth aspects of the atm1 short root phenotype. Our initial discovery of the sugar dependent phenotype was found on media lacking sucrose. We then went on to examine glucose to see if the type of sugar mattered and/or if the short root phenotype was due to sugar sensing or metabolism defect(s). Sugar-mediated growth in Arabidopsis is governed by several distinct, yet overlapping pathways (TOR, HXK, and SnRK) and thus we tried to determine which pathway(s) was of relevance here and how ATM1 may work downstream of such sensors (or not). Sucrose is transported from shoot to root, but is broken down to glucose. Sucrose "sensing" occurs via HXK while TOR and SnRK1 are downstream "energy" sensors which can integrate multiple metabolites (e.g. sugars and amino acids).

We have observed two things in the columella cells of atm1-1 roots: reduced DR5:GFP expression and fewer cell divisions. The cell division defect is consistent with the overall small RAM size. It would be interesting to further characterize the columella abnormalities with auxin production, signaling and/or transport, but such studies would be an additional set of investigations that are beyond the scope of this work.

Comment 1.3: Considering the discussion of auxin, I considered if additional experiments using exogenous auxin would help clarify the interpretation of results, but I think it would be more likely to convolute the data and are likely beyond the scope for this paper, and is unnecessary.

© 2023. Published by The Company of Biologists under the terms of the Creative Commons Attribution License (https://creativecommons.org/licenses/by/4.0/).

Response 1.3: Because auxin treatments lead to inhibition of the primary root, and atm1-1 roots are already small, we did not see the value in doing such exogenous auxin treatments. ATM1 protein, not transcript, levels are regulated by auxin as identified by quantitative proteomics (Kelley et. al., bioRxiv 2017).

Comment 1.4: In some places, class IX (nine) instead of XI (eleven) were written. A find & replace should easily fix this.

Response 1.4: Thank you for pointing out this error, we have fixed this.

Comment 1.5: Regarding ATM1pro:NLS-GFP expression. It would be helpful to have a complementary supplemental data figure showing more confocal images and/or GUS images along the entire root length; it appears that ATM1 expression, based on Figure 1A and 2A likely extends beyond the meristematic region.

Response 1.5: We have added this data as Fig. S2.

Comment 1.6: “Confocal microscopy of GFP-ATM1 in Arabidopsis roots revealed protein accumulation at the plasma membrane..” From the image shown, I don’t think this can be concluded, but I also don’t think it is essential for the conclusions in the paper to definitely show subcellular localization. I recommend “appears ATM is predominantly localized to the cell periphery”. This could be cortical localization, and there is maybe some cytoplasmic localization? The pattern is consistent for myosins, thus I am not overly concerned.

Response 1.6: We have changed this sentence. We have also added additional data showing plasmodesmal localization for ATM1 (Fig. S1). Altogether our expression data for ATM1 are consistent with previous studies based on truncated and/or transient expression of ATM1 (Golomb et. al., BMC Plant Bio 2008 and Haraguchi et. al., J Biol Chem 2014) but represent endogenous, stable expression.

Comment 1.7: Similarly, “Despite the mis-expression of the root cap markers in atm1-1, surprisingly, GFP expression of the QC marker WOX5:GFP is intact the mutant” - it is very hard to see this at the magnification shown. Can more magnified images, where the cells of the QC are clear, be shown? I think given the columella cell phenotype, this was a useful experiment, so clearer images are helpful.

Response 1.7: The expression of the marker is quite clear and is not abnormal or diminished. Also, the cell files in atm1-1 roots are not disordered or absent. We do not think the defects in atm1-1 arise from abnormal QC behavior, but rather a general dampening of cell division in the RAM.

Comment 1.8: Microscopy images should be color-blind friendly by replacing red with magenta. This can be done very quickly in FIJI using Image>color>replace red with magenta or in Photoshop by copy and pasting the red channel into the blue channel.

Response 1.8: We appreciate the need for color barrier free images. Because of the extensive number of images in this study we are not able to re-color the microscopy images at this time.

Comment 1.9: The first paragraph of the transcriptomic analysis reports the number of DEG for different comparisons – there are a lot of pairwise comparisons. This is all in the supplemental data, but it would be very helpful for the first tab in the data to have a table of the DEG between comparisons. Notably, there are relatively few genes different comparing atm1 and WT mock treated, while a more when treated with glc and even more with suc. I think this is helpful to note. The GO enrichment in 5D does not clearly convey this. I think the number of responding genes in the different genotypes/conditions is actually interesting as it (I think?) reflects the phenotypic responses – i.e. greater differences in both phenotype and gene expression are seen in the presence of sucrose, vs untreated.

Response 1.9: The red dots on the volcano plots in Figure 4 indicate that there are many more DE genes in sucrose treated atm1-1 compared to WT (Fig. 4C) in comparison to atm1-1 versus WT (Fig.

© 2023. Published by The Company of Biologists under the terms of the Creative Commons Attribution License (https://creativecommons.org/licenses/by/4.0/).

4A) and glucose treated atm1-1 compared to WT (Fig. 4B). From this data, we observed the greatest alteration in gene expression in sucrose-treated atm1-1 roots compared to wild-type roots treated with sucrose. This is notable because the mutant cannot be fully rescued by sucrose, which suggests that sucrose-dependent gene expression is abormal in atm1-1 roots. The GO enrichment is a way to identify global patterns in which types of genes (or pathways) are collectively altered, and thus can drive new hypotheses. The GO enrichments allowed us to observe that both auxin and cell cycle genes are mis-regulated in atm1-1 roots compared to wild-type. The transcriptomic analyses are extensive and could be examined for many different comparisons; here, we focused on the comparison that was most directly tied to the observed phenotype for which we could follow up with additional experiments.

Comment 1.10: A fair amount of work on myosin VIII had been done in moss, including work on development. Myosin VIII defects lead to a decrease in cell streaming and overall growth, which I think could be mechanistically similar. I think citing these studies is appropriate. Wu, S. Z., Ritchie, J. A., Pan, A. H., Quatrano, R. S., & Bezanilla, M. (2011). Myosin VIII regulates protonemal patterning and developmental timing in the moss Physcomitrella patens. Molecular plant, 4(5), 909-921. Wu, S. Z., & Bezanilla, M. (2014). Myosin VIII associates with microtubule ends and together with actin plays a role in guiding plant cell division. Elife, 3, e03498.

Response 1.10: Thank you for reminding us about the studies on the ATM1 ortholog in Physco, Myosin VIII. We have added these citations to our introduction and discussion sections.

Reviewer 2 Advance Summary and Potential Significance to Field:
The manuscript by Olatunji, Clark and Kelly, characterizes the expression domains of Arabidopsis thaliana Myosin1 (ATM1), a class XII-type myosin, in the root and lateral root meristem and shows that the atm1-1 mutant displays defects in sugar-dependent root and meristem length. The defects can be rescued by expression of GFP-ATM1 under control of its own promoter in the mutant background. The proposed plasma membrane (PM)localization of GFP-ATM1 is not addressed with appropriate markers or sufficient resolution here, but was parly indicated by earlier antibody studies at the electron microscopic level (Reichelt et al., 1999, Plant J.). The authors find the layer of columella stem cell daughter cells lacking in the atm1-1 mutant and interpret this as a defect in the maintenance of CSC identities, which, however is not strictly distinguishable from reduced cell division activity of the columnella stem cells (CSC) by the methods employed. The authors performed a transcriptomic analysis of wild type and the atm1-1 mutant in response to exogenously applied sucrose and glucose and founnd some auxin biosynthesis and response genes were upregulated in the atm1-1 mutant upon sucrose induction, while the auxin response reporter DR5:GFP displayed lower expression in atm1-1 than in the wild type. A mechanistic explanation for this is not provided, nor how this relates to myosin function. Analysis of c5-ethynyl-2-deoxyoridine (EdU) label to detect replicating cells in the root meristem and of a multi-cell-cyle-stage marker revealed a lower number of cells entering S and G2 phase in atm1-1 compared to the wild type. The findings would be of rather specific interest to some plant biologist, but do not convey a mechanistic understanding of ATM1 function in cell cycle progression and with respect to auxin responses and/or biosynthesis,

Reviewer 2 Comments for the Author:
Taken together, while the phenotypic characterization of the atm1-1 mutant is new and the experiments generally thoroughly performed, as far as they go, the study reveals little to no mechanistic insight into via which interactions ATM1 affects cell cycle progression auxin biosynthesis and/or response at the genetic and/or molecular level. This would need to be worked out e.g. by genetic and/or molecular interaction studies in order to gain some mechanistic understanding of ATM1 function.

Response to Reviewer 2:
We have added genetic analyses to this manuscript to further delineate “mechanism” of ATM1 with respect to sugar sensors Hexokinase 1 (Figure 8) and TOR (Figure 9). Our genetic analyses (Figs. 8 & 9) suggest that ATM1 is downstream of TOR. Notably, we were not able to recover atm1 raptor double mutants, presumably to due to embryo lethality and not due to low crossover events as these two genes are not on the same chromosome.

© 2023. Published by The Company of Biologists under the terms of the Creative Commons Attribution License (https://creativecommons.org/licenses/by/4.0/).

Based on the STRING database, ATM1 may interact with ARP2 (Actin-related protein 2), a calmodulin-like protein (CML13) and several exocyst comoplex proteins (SEC6, SEC10, SEC15A, and SEC15B). We have not performed a yeast-two hybrid or other experiment to determine protein partners of ATM1; that would require additional resources for this project which are not available.

Resubmission

First decision letter

MS ID#: DEVELOP/2023/201762
MS TITLE: The Class VIII myosin ATM1 is required for root apical meristem function
AUTHORS: Damilola Olatunji, Natalie M Clark, and Dior Kelley

I have now received the referee's reports on the above manuscript, and have reached a decision. The referee's comments are appended below, or you can access them online: please go to BenchPress and click on the 'Manuscripts with Decisions' queue in the Author Area.

The overall evaluation is positive and we would like to publish a revised manuscript in Development, provided that the referee's comments can be satisfactorily addressed. Please attend to the reviewer's comments in your revised manuscript and detail them in your point-by-point response. If you do not agree with any of their criticisms or suggestions explain clearly why this is so. If it would be helpful, you are welcome to contact us to discuss your revision in greater detail. Please send us a point-by-point response indicating your plans for addressing the referee's comments, and we will look over this and provide further guidance.

Reviewer 1

Advance summary and potential significance to field

This is a revised version of a paper examining the role of a myosin XIII protein, ATM1, and its relationship to growth, sugar metabolism/signaling, and auxin. Both sugar signaling and hormone signaling are very complex pathway that somehow interconnect into downstream outputs (i.e. growth). This manuscript uses a myosin mutant to understand how these pathways may interact. Recently, myosin proteins have been garnering attention in developmental and growth processes (e.g. Han et al., 2021 as cited in the MS), and therefore this manuscript is topical.

Comments for the author

In my opinion, the double mutant analysis with gin1 and TOR OE greatly increases the strength of this paper. It also helps contextualize the transcriptomic data. In my opinion, no further experiments are necessary. I encourage the authors to consider amending the text/conclusions, as noted below.

1. gin1 data
I am a bit confused about/disagree with the way the conclusions are presented.
Lines 314-316: "The gin2 atm1-1 double mutant root growth phenotype does not show any additive epistatic interaction, but is the same as the parental single mutants. This suggests that ATM1 may be downstream of the hexokinase 1 pathway."

-I agree with this conclusion (technically, ATM1 could also be upstream, but I think based on protein function that is unlikely).
Lines 335-336 "these genetic analyses imply that HXK1 is not a key player in ATM1- mediated root growth"
-I don't see how that is true. If a gene/protein is downstream of another in the same pathway, how is that not a "key player"? Similarly, the title for "Fig. 8. ATM1 activity is independent on Hexokinase 1 sugar signaling pathway." seems incorrect, if ATM1 is downstream. I think the data

© 2023. Published by The Company of Biologists under the terms of the Creative Commons Attribution License (https://creativecommons.org/licenses/by/4.0/).

are solid and very helpful, but advise rewording. E.g., “Fig 8. Genetic analysis indicates no genetic interactions between atm1 and the hexokinase sensor gin1.” (or similar, more neutral statement.) Similarly, changing lines 335 to something more akin to line 314.

2. The model in the final figure has changed, but I am a bit lost. LST8 and ROP2 are both included and discussed no where in the text. Experiments are unnecessary, but where did they come from? Are there relevant citations? Please contextualize the model better.

3. I still think a few sentences in the paper regarding the impetus for sucrose vs glucose experiments in the first place (i.e., why did you do both sugars, and not just sucrose, for example) to help the reader would strengthen the manuscript.

I want to note a couple of significant improvements from last time. -Previously, I had a minor concern regarding the plasma membrane localization, as I felt the low magnification image made it hard to conclude (although this is consistent with previous observations). With the supplemental transient expression, I am no longer concerned. I would also like to acknowledge how difficult it can be to obtain stable transgenics that complement the phenotype for a myosin protein. They are large proteins difficult to work with.
-The GUS expression in figure S1 is very helpful.

First revision

Author response to reviewers' comments

DEVELOP/2023/201762

Response to reviewers 4-17-2023

Reviewer 1 Advance summary and potential significance to field
This is a revised version of a paper examining the role of a myosin XIII protein, ATM1, and its relationship to growth, sugar metabolism/signaling, and auxin. Both sugar signaling and hormone signaling are very complex pathway that somehow interconnect into downstream outputs (i.e. growth). This manuscript uses a myosin mutant to understand how these pathways may interact. Recently, myosin proteins have been garnering attention in developmental and growth processes (e.g. Han et al., 2021 as cited in the MS), and therefore this manuscript is topical.

Reviewer 1 Comments for the author

In my opinion, the double mutant analysis with gin1 and TOR OE greatly increases the strength of this paper. It also helps contextualize the transcriptomic data. In my opinion, no further experiments are necessary. I encourage the authors to consider amending the text/conclusions, as noted below.

Response from authors: Thank you for the review of the revised manuscript and for providing additional feedback. We have addressed each comment (1-3) below and tracked changes in the revised manuscript.

Comment 1: gin1 data. I am a bit confused about/disagree with the way the conclusions are presented. Lines 314-316: “ The gin2 atm1-1 double mutant root growth phenotype does not show any additive epistatic interaction, but is the same as the parental single mutants. This suggests that ATM1 may be downstream of the hexokinase 1 pathway.”
-I agree with this conclusion (technically, ATM1 could also be upstream, but I think based on protein function that is unlikely).
Lines 335-336 “these genetic analyses imply that HXK1 is not a key player in ATM1-mediated root growth”

© 2023. Published by The Company of Biologists under the terms of the Creative Commons Attribution License (https://creativecommons.org/licenses/by/4.0/).

-I don't see how that is true. If a gene/protein is downstream of another in the same pathway, how is that not a "key player"? Similarly, the title for "Fig. 8. ATM1 activity is independent on Hexokinase 1 sugar signaling pathway." seems incorrect, if ATM1 is downstream. I think the data are solid and very helpful, but advise rewording. E.g., "Fig 8. Genetic analysis indicates no genetic interactions between atm1 and the hexokinase sensor gin1." (or similar, more neutral statement.) Similarly, changing lines 335 to something more akin to line 314.

Response 1: We have revised the results and interpretation regarding the atm1 gin2 double mutant (lines 316-321) and the Figure 8 legend (lines 716-722).

Comment 2.The model in the final figure has changed, but I am a bit lost. LST8 and ROP2 are both included and discussed no where in the text. Experiments are unnecessary, but where did they come from? Are there relevant citations? Please contextualize the model better.

Response 2: We have added additional text in the manuscript to clarify the inclusion of LST8 in the TOR COMPLEX (lines 78-82) and removed ROP2 from the model. LST8 is a conserved part of the TOR complex and thus is included in the model, but its role is currently unknown (in any eukaryote). The model has been clarified via additional text (lines 460-463) and within the Figure 10 legend (lines 738-745).

Comment 3.I still think a few sentences in the paper regarding the impetus for sucrose vs glucose experiments in the first place (i.e., why did you do both sugars, and not just sucrose, for example) to help the reader would strengthen the manuscript.

Response 3: We have added additional text about the justification of examining responses to both glucose and sucrose throughout the manuscript (lines 221-225, lines 305-321).

I want to note a couple of significant improvements from last time.
-Previously, I had a minor concern regarding the plasma membrane localization, as I felt the low magnification image made it hard to conclude (although this is consistent with previous observations). With the supplemental transient expression, I am no longer concerned.
I would also like to acknowledge how difficult it can be to obtain stable transgenics that complement the phenotype for a myosin protein. They are large proteins difficult to work with.
-The GUS expression in figure S1 is very helpful.

Response from authors: Thank you for the feedback on the ATM1 subcellular localization and the GUS reporter data. We are glad that we were able to address these concerns and clarify the ATM1 expression patterns.

Second decision letter

MS ID#: DEVELOP/2023/201762

MS TITLE: The Class VIII myosin ATM1 is required for root apical meristem function

AUTHORS: Damilola Olatunji, Natalie M Clark, and Dior Kelley
ARTICLE TYPE: Research Article

I am happy to tell you that your manuscript has been accepted for publication in Development, pending our standard ethics checks.

Reviewer 1

Advance summary and potential significance to field

Please see previous reveiws.

© 2023. Published by The Company of Biologists under the terms of the Creative Commons Attribution License (https://creativecommons.org/licenses/by/4.0/).

Comments for the author

All my oncerns have been addressed. My only minor comment is in the discussion regarding the model, this sentence does not make sense to me:

"Because the overexpression of TOR kinase could not restore atm1-1 short phenotype could further suggest that TOR is a hub of energy integration and not an activator of sugar molecules"

It seems odd - what does "activator of sugar molecules" mean?

© 2023. Published by The Company of Biologists under the terms of the Creative Commons Attribution License (https://creativecommons.org/licenses/by/4.0/).
